# Sensory-based non-pharmacological interventions for reducing dental anxiety and improving behavioural cooperation in paediatric patients: a systematic review with GRADE assessment

**DOI:** 10.3389/fdmed.2026.1895336

**Published:** 2026-10-14

**Authors:** Drishadvati Mukherjee, Swagata Saha, Aishani Baksi, Manju Raman Nair

**Affiliations:** 1 Department of Paediatric and Preventive Dentistry, A B Shetty Memorial Institute of Dental Sciences, NITTE (Deemed to be University), Mangaluru, Karnataka, India; 2 Department of Pedodontics and Preventive Dentistry, Dr. D Y Patil Dental College & Hospital, Dr. D Y Patil Vidyapeeth (Deemed to be University), Pune, Maharashtra, India

**Keywords:** behaviour management, dental anxiety, grade, paediatric dentistry, sensory-based interventions, systematic review, virtual reality

## Abstract

**Background and objectives:**

Dental anxiety affects an estimated 9%–20% of children and disproportionately impacts those with neurodevelopmental conditions. Sensory-based non-pharmacological interventions, which intentionally modulate auditory, visual, tactile, olfactory, or proprioceptive input, have emerged as promising approaches for anxiety and behaviour management in paediatric dentistry, but no published review has applied formal GRADE certainty assessment across multiple sensory-based modalities. This review evaluated the effectiveness of sensory-based interventions for dental anxiety and behavioural cooperation in paediatric patients (≤18 years) and assessed certainty of evidence using GRADE.

**Eligibility criteria:**

RCTs and controlled clinical trials involving paediatric participants (≤18 years) evaluating sensory-based interventions delivered during dental treatment or as pre-visit preparation, reporting dental anxiety, behavioural cooperation, or physiological stress, were eligible for GRADE assessment; eligible non-randomised, observational, pilot, feasibility, descriptive, and secondary studies were retained for narrative synthesis only. Studies of exclusively pharmacological or non-sensory cognitive-behavioural interventions were excluded.

**Methods:**

MEDLINE (PubMed), Scopus, Embase, PsycINFO, and CENTRAL were searched from inception to 27 July 2026, supplemented by manual reference screening and Google Scholar searches. Two reviewers independently performed study selection, data extraction, risk-of-bias assessment (RoB 2), and GRADE assessment. Given substantial heterogeneity, findings were synthesised narratively.

**Results:**

Of 2,002 records identified, 47 studies met inclusion criteria: 25 RCTs/controlled crossover trials contributing to GRADE assessment, and 22 non-randomised, pilot, observational, or secondary studies retained for narrative synthesis. Nine modalities were evaluated: sensory-adapted dental environments (SADE), audiovisual distraction/virtual reality (VR), music therapy, aromatherapy, weighted blankets, social stories, video modelling, visual pedagogy, and emerging sensory-based approaches. SADE, audiovisual distraction/VR, and music therapy showed the most consistent improvements in anxiety, cooperation, physiological stress, and pain, supported by moderate-certainty evidence for SADE and audiovisual distraction/VR; for music therapy, the direct paediatric primary-trial base is now a single GRADE-eligible RCT following reclassification of Chen and Hawkins (2021) to Tier 2 as a single-arm pilot without a concurrent comparator; certainty for music-therapy anxiety reduction is correspondingly rated low, supported by corroborating secondary meta-analyses. Certainty across modalities ranged from very low to moderate, limited by risk of bias, heterogeneity, inconsistent outcome measures, and imprecision.

**Conclusions:**

Sensory-based interventions show promising benefit for dental anxiety and behavioural cooperation in paediatric dental care, with the strongest (moderate-certainty) evidence for SADE and audiovisual distraction/VR, and low-to-moderate-certainty evidence for music therapy. Overall certainty remains low to moderate, supporting cautious, individualised clinical use rather than routine first-line implementation. Adequately powered trials with standardised outcomes, prospective registration, and GRADE-informed reporting are needed to strengthen the evidence base.

**Systematic Review Registration**: identifier CRD420261456366.

**Table 1:** Characteristics of the 47 included studies, grouped by intervention modality.

Author (Year)	Country/Design	Population	*N*	Intervention vs. comparator	Main findings	Evidence tier
SADE — Sensory-Adapted Dental Environments						
Shapiro et al. (2007) ( 3)	Israel — Quasi-experimental controlled (crossover; not RoB2-assessed)	Neurotypical children	35 (16 IDD + 19 TD)	SADE (dimmed lighting, calming stimuli) vs. conventional environment	Anxiety episode duration/intensity ↓ (*p* < 0.01); cooperation ↑ (*p* = 0.004); episode frequency unchanged; lower EDA in SADE	Tier 1 (GRADE)
Stein Duker et al. (2023) ( 6)	USA — RCT (crossover)	Children with ASD	138	Comprehensive multisensory SADE vs. conventional operatory	Lower SCL (−1.22 μS); distress reduced (d = −0.84 to −1.19); no adverse effects	Tier 1 (GRADE)
Mehta et al. (2024) ( 7)	India — RCT	Children with Down syndrome	40 (20/20)	SADE waiting-room adaptation vs. standard waiting area	HR and Venham anxiety significantly lower with SADE (*p* ≤ 0.03)	Tier 1 (GRADE)
Gupta et al. (2025) ( 8)	India — Observational (comparative)	Potentially cooperative children	40	SADE vs. standard dental environment	Higher Frankl scores in SADE; strong parental preference	Tier 2 (narrative)
Kaintura et al. (2024) ( 9)	India — Descriptive/mechanistic	Children with ASD	3 (case series)	Personalised SADE guided by EDA monitoring	Mechanistic insight into individual physiological response	Tier 2 (narrative)
Panigrahi & Mohapatra (2026) ( 13)	India — Controlled clinical study	Children with ASD (pulp therapy)	60 (30/30)	Multisensory environmental adaptation vs. conventional treatment environment	Cooperation 4.6 ± 0.3 vs. 3.2 ± 0.5 (*p* < 0.001); anxiety 2.1 ± 1.0 vs. 5.8 ± 1.2 (*p* < 0.001); completion 93% vs. 67% (*p* = 0.01)	Tier 2 (narrative)
Veseli et al. (2025) ( 57)	Kosovo — Secondary narrative synthesis	ASD, Down syndrome, neurotypical children	NR	Multisensory dental environments (synthesis)	Consistent behavioural improvement; feasible for routine implementation	Tier 2 (narrative, secondary)
Rathi & Saha (2025) ( 14)	India — Conceptual/descriptive proposal	—	—	CIPE-NRT© immersive environment concept	Proposed future immersive multisensory environmental design	Tier 2 (narrative)
Fathima et al. (2024) ( 42)	India — Pilot, parallel-arm interventional (randomisation unconfirmed)	Neurotypically healthy children	148	SADE vs. regular dental environment (RDE)	No baseline group difference; SADE group improved behaviour and anxiety; RDE group improved behaviour only	Tier 2 (narrative)
Audiovisual Distraction and Virtual Reality (VR)						
Alshatrat et al. (2025) ( 15)	Jordan — RCT	Children undergoing dental procedures	154 (77/77)	Immersive VR vs. conventional care (no VR)	Lower pain (*p* < 0.05); 70.31% reported relaxation; no adverse events	Tier 1 (GRADE)
Hamdy et al. (2024) ( 44)	Egypt — RCT (3-arm)	Children	45 (15/15/15)	Active VR/Passive VR vs. standard dental care	Both VR arms reduced pain, anxiety and heart rate vs. control	Tier 1 (GRADE)
Fallea et al. (2025) ( 16)	Italy — RCT	Children with ASD	64	VR-based oral-hygiene education vs. traditional instruction	OHAT 7.6 ± 1.8 vs. 3.3 ± 1.4 (t(62) = 10.4; *p* < 0.001); higher engagement	Tier 1 (GRADE)
Aly et al. (2025) ( 10)	Egypt — RCT (3-arm)	Children with ADHD	48	VR glasses/white-noise distraction vs. no-distraction control	Both VR and white noise better than control (*p* = 0.005); white noise more consistent physiologically	Tier 1 (GRADE)
Üstün et al. (2025) ( 17)	Turkey — RCT (crossover)	Children (mean age 6.69 ± 1.08y)	68	Cartoon-assisted audiovisual distraction vs. tell-show-do	Pain reduced (*p* < 0.025) and cooperation improved; anxiety not significantly different	Tier 1 (GRADE)
Mohammadi et al. (2025) ( 50)	Iran — RCT	Children 6–12y	36 (18/18)	VR-based distraction vs. no-VR control	Anxiety lower (13.89 ± 1.78 vs. 21.22 ± 3.75; *p* < 0.001); HR rose less with VR (*p* < 0.001)	Tier 1 (GRADE)
Kasimoglu et al. (2025)	Turkey — RCT (3-arm, parallel)	Children 6–10y (pulpotomy)	90 (30/30/30)	Active VR-glasses/passive cartoon distraction vs. no-distraction control	Active VR most favourable post-treatment FIS (1.30 vs. 1.47 vs. 1.63)	Tier 1 (GRADE)
Bali et al. (2025) ( 37)	India — RCT	Children 6–13y (cavity preparation)	40 (20/20)	Smartphone music + audioanalgesia vs. basic behavioural guidance	Anxiety improved more (*p* < 0.001); Venham (*p* = 0.158) and FLACC (*p* = 0.22) not significant	Tier 1 (GRADE)
Pisano et al. (2024) ( 53)	Italy — Secondary synthesis	Children with special healthcare needs	11 studies	Immersive VR/audiovisual glasses vs. tablet-based distraction	VR/AV glasses generally more favourable than tablet distraction	Tier 2 (narrative, secondary)
Trusculescu et al. (2025)	Romania — Pilot, within-subject (baseline vs. VR)	Children 6–12y	120	Immersive relaxing VR (beach/forest/underwater) vs. own pre-VR baseline	Reduced heart rate and perspiration during VR exposure	Tier 2 (narrative)
Music Therapy and Auditory Distraction						
Kuppusamy et al. (2025)	India — RCT (3-arm)	Orphaned children 12–15y	80 (25/29/26)	Musical drama/music listening vs. tell-show-do	Anxiety reduced 15.32 ± 6.40 and 15.06 ± 7.62 vs. 4.00 ± 4.29 with tell-show-do (*p* < 0.001)	Tier 1 (GRADE)
Chen & Hawkins (2021) ( 40)	USA — Controlled study — single-arm pilot; no concurrent comparator group reported	Children with intellectual/developmental disability	15 (pilot, no comparator)	Pre-procedural music listening vs. usual care (historical/before-after, not a concurrent comparator)	Anxiety (*p* = 0.001) and pulse rate (*p* = 0.02) reduced vs. baseline; reduced premedication requirement	Tier 2 (narrative) — RECLASSIFIED from Tier 1. Rationale: Section 2.2 requires a concurrent comparator group for Tier 1 (GRADE) eligibility; this study is a single-arm pilot without one and is therefore retained for contextual/narrative synthesis only.
Marqués-Martínez et al. (2025) ( 49)	Spain — Systematic review & meta-analysis	Children 4–14y	476 (7 RCTs)	Music-based interventions vs. standard care	SMD −0.48 (95% CI −0.72 to −0.25; *p* < 0.001), I²=42%; HR SMD −0.42 (*p* = 0.002)	Tier 2 (narrative, secondary)
López-Valverde et al. (2024) ( 19)	Spain/Portugal — Systematic review & meta-analysis	Mixed, with paediatric subgroup	14 RCTs	Music therapy vs. standard care	Significant overall effect (*p* = 0.005); paediatric subgroup effect *p* < 0.00001	Tier 2 (narrative, secondary)
Aromatherapy						
Abdalhai et al. (2024) ( 11)	Syria — RCT	Children with dental anxiety	56 (28/28)	Lavender–neroli aromatherapy + music vs. control	Anxiety, HR, BP significantly better (*p* < 0.05); pain not significant (*p* = 0.176)	Tier 1 (GRADE)
Janthasila & Keeratisiroj (2023) ( 46)	Thailand — RCT (4-arm)	Children	128 (32/33/31/32)	Music/aromatherapy/combined vs. control	Combined music + aromatherapy most favourable (F = 22.22; *p* < 0.001)	Tier 1 (GRADE)
Bagaria et al. (2026) ( 20)	India — RCT (3-arm)	Paediatric dental patients	45	Aromatherapy + relaxation music/visual distraction vs. conventional care	Both intervention arms had significantly lower anxiety and FLACC scores than control	Tier 1 (GRADE)
Bartakke et al. (2025) ( 38)	India — RCT	Children 6–10y, first dental visit	64	Lavender-oil aromatherapy vs. bubble-breath play therapy (BBPT)	Both reduced anxiety; BBPT > aromatherapy (*p* < 0.001); BBPT also improved pulse (*p* < 0.05) and SpO2 (*p* < 0.05)	Tier 1 (GRADE)
Weighted Blankets and Deep-Pressure Interventions						
Ajgaonkar et al. (2026) ( 36)	India — RCT (crossover)	Children 6–14y with mild IDD	49 (26/23)	Weighted blanket (∼10% body weight) vs. tell-show-do (no blanket)	Anxiety reduced in one sequence group (*p* = 0.0031); BP/pulse favoured blanket; SpO2 improved (+2.99%; *p* = 0.001)	Tier 1 (GRADE)
Alfailkawi et al. (2026) ( 21)	USA — RCT	Children under N2O sedation	77	Weighted stuffed animal vs. standard care	Frankl median 4 vs. 3 (*p* < 0.001); anxiety lower (*p* = 0.02); no adverse events	Tier 1 (GRADE)
Stein Duker et al. (2025) ( 22)	USA — Feasibility/acceptability study	Children (non-invasive dental care)	20 children/20 caregivers/9 dentists	Weighted blanket (implementation study)	Mean scores >4.70 (caregiver, child, clinician); well tolerated	Tier 2 (narrative)
McBeain et al. (2022) ( 23)	USA — Retrospective comparative	Children (moderate-sedation visits)	125 sedation cases	Weighted blanket availability vs. pre-introduction period (protective stabilisation)	Use declined 78.7%→32.8%; adjusted OR 0.067 (95% CI 0.020–0.22; *p* < 0.001)	Tier 2 (narrative, reclassified from Tier 1)
Social Stories						
Zhou et al. (2020) ( 24)	Hong Kong — RCT (24-month follow-up)	Preschool children with special healthcare needs	∼306 (approx.; exact RCT N not independently confirmed)	Social story-based oral-health promotion vs. standard care	Attendance OR 2.00 (95% CI 1.15–3.49); no significant difference in caries at 24 months	Tier 1 (GRADE)
Gandhi et al. (2024) ( 25)	USA — Pilot comparative study	Children with ASD	Not independently confirmed (pilot)	Social stories vs. video modelling (head-to-head)	Both improved oral hygiene; no significant between-group difference; caregivers preferred video modelling	Tier 2 (narrative)
Zafar et al. (2025) ( 58)	Australia — Clinician survey	Dental specialists managing ASD	Not stated (62% response rate)	Social stories (perceived utility)	72% overall endorsement; 94% (level 1 ASD) to 44% (level 3) (*p* < 0.05)	Tier 2 (narrative)
Video Modelling						
da Silva Moro et al. (2024; DOI 2022 online-first) ( 26)	Brazil — RCT	Children with ASD	Not independently confirmed	Video modelling vs. standard care	Greater proportion of planned dental-care steps completed (*p* < 0.05)	Tier 1 (GRADE)
Peker et al. (2026) ( 27)	Turkey — Pre-post interventional	Children with ASD	12	POV video modelling + systematic desensitisation vs. pre-intervention baseline	Pulse 98.16 ± 16.06 → 80.33 ± 11.15 bpm (*p* = 0.013); Frankl improved (*p* < 0.05)	Tier 2 (narrative)
Hine et al. (2019) ( 28)	USA — Pre-post interventional	Children (first dental visit)	40	Dentist-created preparatory video vs. pre-intervention baseline	Reduced disruptive behaviour; improved cooperation	Tier 2 (narrative)
Popple et al. (2016)	USA — Pilot/feasibility study	Children with ASD	18	Remote video modelling (oral hygiene)	Feasible delivery; improved oral-hygiene-related behaviour	Tier 2 (narrative)
Visual Pedagogy and Visual Schedule Systems						
Nilchian et al. (2017) ( 29)	Iran — RCT (double-blind)	Children with ASD	40	Visual pedagogy vs. control	Greater cooperation during fluoride therapy (*p* ≤ 0.001); not significant for several other procedures	Tier 1 (GRADE)
Cirio et al. (2022) ( 30)	Italy — RCT	Children with ASD	84	Video-based visual supports vs. photograph-based visual aids	More examination steps completed with video-based supports (*p* = 0.04)	Tier 1 (GRADE)
Du et al. (2022) ( 41)	Hong Kong — Controlled interventional	Preschool children with ASD	122	Structured visual toothbrushing programme vs. comparison group	Lower plaque/gingival scores at 3 and 6 months vs. baseline and comparator	Tier 2 (narrative)
Mah & Tsang (2016) ( 31)	Canada — Pilot study	Children attending dental appointments	14	Visual schedule system vs. tell-show-do	+1.38 additional steps; −35.52 s/step; −18.7% distress	Tier 2 (narrative)
Raja et al. (2025) ( 32)	India — RCT (head-to-head)	Children with ASD	72 (36/36)	Music & movement therapy vs. visual pedagogy	Music/movement therapy superior to visual pedagogy alone	Tier 2 (narrative)
Emerging and Novel Sensory Modalities						
Kasimoglu et al. (2020) ( 33)	Turkey — RCT	Children	200 (100/100)	Social robot (interactive assistant) vs. conventional management	Lower pulse rate and FIS (*p* < 0.05); 88.3% preferred robot presence at future visits	Tier 1 (GRADE)
Sreenivasan et al. (2025) ( 34)	India — Comparative clinical evaluation	Children with ASD and healthy uncooperative children	34 (17/17)	Snoezelen multisensory environment	Significant reductions in both groups; more pronounced among children with ASD	Tier 2 (narrative)
Ramassamy et al. (2019) ( 12)	India — Controlled interventional	Children with ASD	72 (36/36)	Yoga therapy (adjunct) vs. toothbrushing instruction alone	Lower plaque/gingival index at 2, 3 and 6 months	Tier 2 (narrative)
Kashyap et al. (2025) ( 47)	India — RCT (3-arm)	Children 6–12y	90 (30/30/30)	Animal-assisted therapy/VR vs. playroom-activity behaviour modification	SEM differed across all groups (*p* < 0.01); both AAT and VR reduced anxiety vs. control	Tier 1 (GRADE)

The Chen and Hawkins ( 40) row (Music Therapy group) has been corrected in this revision: the Evidence Tier is changed from “Tier 1 (GRADE)” to “Tier 2 (narrative)”, with an explanatory note added directly in the table, because the study is a single-arm pilot without a concurrent comparator group and therefore does not meet the Tier 1 definition in Section 2.2. See also the Author Response Letter, Item 1.

## 1 Introduction

Dental anxiety affects an estimated 9%–20% of children and remains one of the most significant barriers to effective oral healthcare delivery in paediatric dentistry (1). Its prevalence is substantially higher among children with neurodevelopmental conditions—including autism spectrum disorder (ASD), attention-deficit/hyperactivity disorder (ADHD), intellectual and developmental disabilities, and Down syndrome—where difficulties in communication, behavioural flexibility, and sensory processing frequently compound anxiety and reduce cooperation during dental care (2). Poorly managed dental anxiety contributes to treatment avoidance, poorer oral health outcomes, and the persistence of negative dental experiences into adulthood.

The dental clinic is a complex sensory environment—unfamiliar sounds, bright lighting, tactile stimulation, distinctive odours, and unpredictable procedures—that can provoke physiological and behavioural stress responses even in neurotypical children (3). For children with sensory processing differences, reported in approximately 69%–95% of individuals with ASD, these demands may become overwhelming. Pharmacological approaches, including nitrous oxide sedation (4) and general anaesthesia, remain effective for severe anxiety but carry increased cost, resource requirements, and potential adverse effects, limiting their suitability as routine strategies. There is therefore a clear clinical need for safe, accessible, developmentally appropriate non-pharmacological interventions suited to everyday paediatric practice.

Sensory-based non-pharmacological interventions—which intentionally modify sensory input across auditory, visual, tactile, olfactory, and proprioceptive pathways to minimise environmental stressors and facilitate emotional and behavioural regulation—have emerged as an increasingly investigated approach (3). The literature encompasses sensory-adapted dental environments (SADE), audiovisual distraction and virtual reality (VR), music therapy, aromatherapy, deep-pressure interventions, visual pedagogy, social stories, video modelling (5), and other emerging strategies. These interventions differ substantially in theoretical basis, implementation, target population, and outcome measures, producing considerable methodological heterogeneity.

Previous evidence syntheses have largely focused on single modalities or specific populations; some have incorporated risk-of-bias assessment and GRADE methodology, but no published review has comprehensively synthesised and compared effectiveness and certainty of evidence across the full spectrum of sensory-based modalities within one evidence framework. Clinicians, researchers, and guideline developers therefore lack a quality-appraised, cross-modality synthesis to inform evidence-based selection of sensory interventions according to patient characteristics and clinical context.

Accordingly, this systematic review aimed to evaluate the effectiveness of sensory-based non-pharmacological interventions in reducing dental anxiety and improving behavioural cooperation among paediatric patients aged 18 years or younger, and to assess the certainty of the available evidence using the GRADE framework, so as to provide clinicians with a comprehensive, quality-appraised evidence base and to identify priorities for future research.

## 2 Methods

### 2.1 Study design and reporting

This review was conducted in accordance with the PRISMA 2020 statement (Page et al., 52). The protocol was developed *a priori* and registered with PROSPERO (CRD420261456366); registration was completed concurrently with manuscript submission rather than prospectively, which is acknowledged as a limitation in the Discussion. Certainty of evidence was evaluated using the GRADE framework, and methodological quality was assessed using the Cochrane Risk of Bias 2 (RoB 2) tool where applicable. The PRISMA 2020 checklist is provided as Supplementary File S1.

### 2.2 Eligibility criteria

Eligibility was defined *a priori* using the PICOS framework.

Population: Children and adolescents ≤18 years undergoing dental treatment or structured preparation for dental care, including both neurotypical individuals and those with neurodevelopmental or developmental conditions (ASD, ADHD, intellectual and developmental disability, Down syndrome, sensory processing differences). Neurodevelopmental status was recorded at extraction but not applied as a formal stratification variable during synthesis (addressed as a limitation in the Discussion). Adult-only studies were excluded; mixed adult–paediatric studies were eligible only when paediatric data could be fully disaggregated.

Intervention: Sensory-based, non-pharmacological strategies whose primary mechanism directly modified or regulated sensory input (auditory, visual, tactile, olfactory, or proprioceptive). Interventions acting primarily through indirect autonomic or neuromodulatory pathways were eligible only where a direct, patient-perceptible sensory-input component was also present; on this basis, auricular acupuncture (direct tactile/pressure stimulation) and animal-assisted therapy (direct multimodal sensory change) were retained. Interventions based exclusively on behavioural, cognitive, communication, or pharmacological approaches were excluded.

Comparator: Standard dental care, an alternative active sensory-based intervention, sham/placebo, attention-control, or other comparator groups typical of controlled clinical research. Studies without a comparator were excluded from the primary effectiveness synthesis and, consistent with this criterion, single-arm pilot studies without a concurrent comparator group do not meet Tier 1 (GRADE) eligibility and are retained as Tier 2 (narrative) evidence — applied on re-review to Chen and Hawkins (40; Table 1, Section 3.4.3), which was a single-arm pre–post pilot with no concurrent comparator group.

Outcomes: Primary outcomes were dental anxiety, behavioural cooperation, and physiological indicators of stress (heart rate, blood pressure, skin conductance, heart-rate variability). Secondary outcomes included pain, procedure completion, oral health-related outcomes, caregiver satisfaction, treatment acceptability, premedication requirements, and adverse events.

Study Design: A two-tier framework distinguished evidence contributing to certainty assessment from contextual evidence. Tier 1 (GRADE-eligible) comprised RCTs and controlled clinical trials (including crossover designs) with a concurrent comparator group in paediatric populations. Tier 2 (narrative) comprised non-randomised controlled, observational, pre-post, pilot/feasibility, descriptive, and secondary studies (systematic reviews/meta-analyses), included for contextual and mechanistic insight but not entered into formal GRADE assessment.

No language restriction was applied during searching, though all studies ultimately meeting eligibility were published in English. Conference abstracts, editorials, letters, narrative reviews, opinion articles, protocols, dissertations, case reports, and unpublished studies were excluded.

### 2.3 Information sources and search strategy

The search was conducted per the Cochrane Handbook (Higgins et al., 45) and PRISMA 2020 (Page et al., 52). Five databases—MEDLINE (PubMed), Scopus, Embase, PsycINFO, and CENTRAL—were searched from inception to 27 July 2026, combining controlled vocabulary (MeSH/Emtree) with free-text terms across three concepts: paediatric dental care, sensory-based/sensory-modulation interventions, and paediatric/neurodevelopmental populations. Full strategies are in Supplementary File S2.

Searching occurred in two stages: an initial search to March 2025 (original protocol), and an update from March 2025 to 27 July 2026 to capture newly published evidence; results were combined into a single reconciled evidence base with duplicates removed before screening. Per-stage yields are in Supplementary File S2; consolidated screening results are in the PRISMA 2020 flow diagram (Figure 1). Supplementary searching included manual reference-list screening of included studies and relevant reviews, and Google Scholar searching restricted to the first 200 results per query (Haddaway et al., (43); full queries in Supplementary File S2, Table S2.3). Records were exported for duplicate removal and imported into Rayyan (Ouzzani et al., 51) for blinded title/abstract screening. Searches were restricted to peer-reviewed published studies; conference abstracts, protocols, dissertations, editorials, reviews, letters, and unpublished/ongoing studies without peer-reviewed outcome data were excluded but examined for potentially eligible published reports.

**Figure 1:**
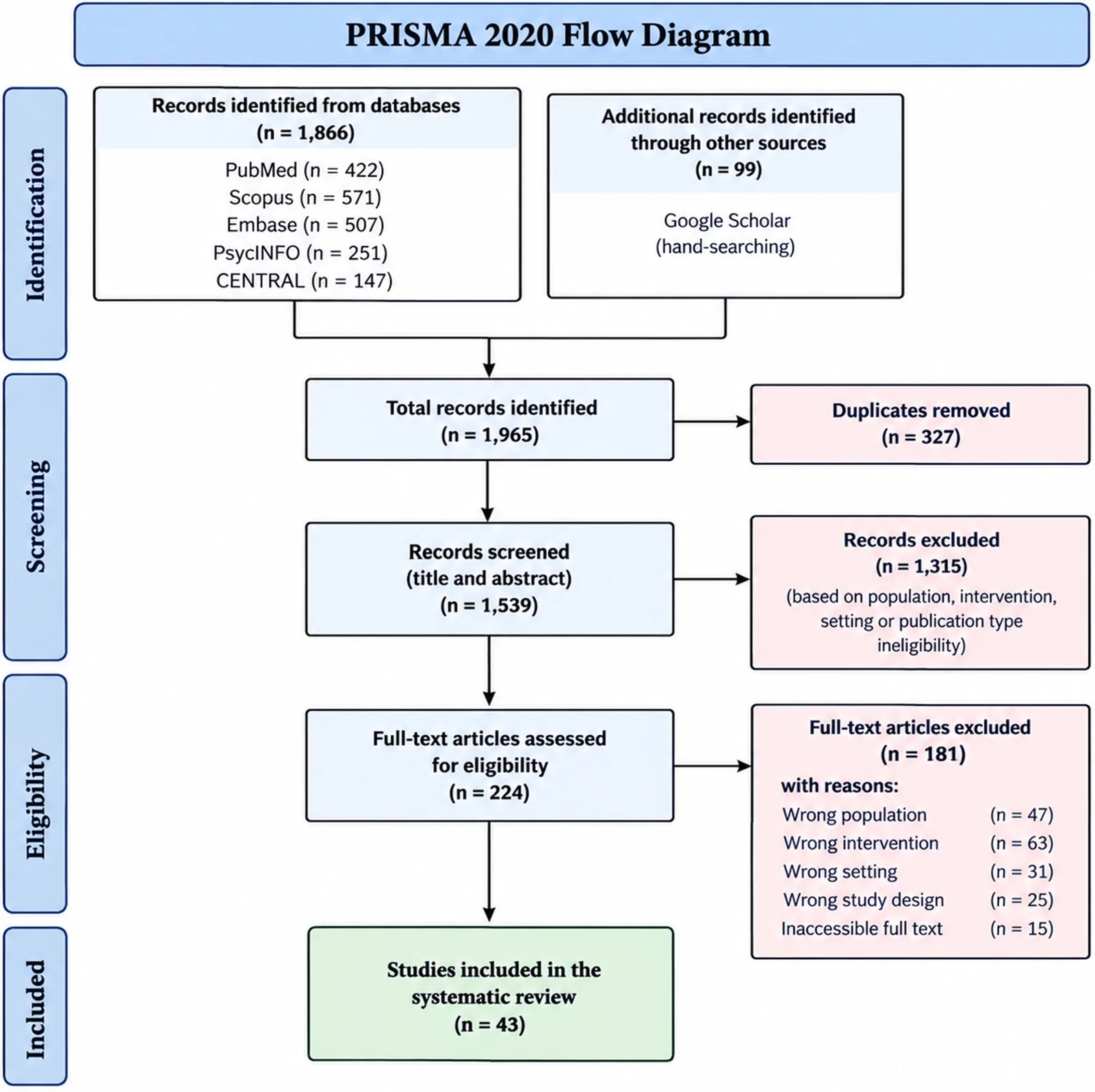
PRISMA 2020 flow diagram. [The “Reports excluded (*n* = 192)” total in the diagram is corrected to sum exactly to its own listed reasons (53 + 64 + 32 + 26 + 17 = 192), replacing the previous internally inconsistent total of *n* = 191 — see Section 3.1 and the Author Response Letter, Item 2.].

### 2.4 Study selection

Screening proceeded in two stages (title/abstract; full-text) following duplicate removal, using Rayyan for blinded, independent review. Two reviewers (D.M., S.S.) first piloted a calibration sample of 50 records (Cohen's *κ* = 0.79) before formal screening. Participant age was verified directly from source publications for every potentially eligible study; mixed-age studies were included only when paediatric data could be extracted separately. Reasons for full-text exclusion were documented individually by predefined category (Supplementary File S3). Disagreements were resolved by discussion, with a third reviewer (M.R.N.) adjudicating where necessary. Inter-reviewer agreement was substantial at title/abstract screening (*κ* = 0.82; 95% CI: 0.74–0.90; 37 disagreements, none requiring adjudication) and near-perfect at full-text assessment (*κ* = 0.88; 95% CI: 0.79–0.97; 9 disagreements, 2 adjudicated). The full selection process is shown in the PRISMA 2020 flow diagram (Figure 1).

### 2.5 Data extraction

Data were extracted using a standardised, pilot-tested form (piloted on five studies by D.M. and S.S.), capturing bibliographic details; study design, setting, and sample characteristics (including age and neurodevelopmental status); intervention characteristics; outcome measures and assessment methods; principal findings; and information relevant to risk-of-bias and certainty assessment. A mapping of outcome measures and instruments used across studies is in Supplementary File S4. For crossover studies, sequence, washout, and carry-over information were extracted to support risk-of-bias assessment. Secondary evidence (systematic reviews/meta-analyses) was appraised using AMSTAR 2 (Shea et al., 55; Supplementary File S5). Extracted data were compared for consistency, with discrepancies resolved by discussion or, where unresolved, third-reviewer adjudication (M.R.N.), re-examining source publications as needed.

### 2.6 Risk of bias assessment

Eligible RCTs and controlled crossover trials were independently assessed by two reviewers (D.M., S.S.) using Cochrane RoB 2 (Sterne et al., 56) across five domains: randomisation process; deviations from intended interventions; missing outcome data; measurement of the outcome; and selection of the reported result. For crossover trials, inadequate or unreported washout was treated as a potential source of bias under Domain 2 and, where outcomes were susceptible to residual carry-over, Domain 4 (resulting judgements in Supplementary File S6, reflected in Table 2). Quasi-experimental studies not meeting RoB 2 assumptions were included but assessed narratively rather than formally. Disagreements were resolved by discussion or third-reviewer adjudication (M.R.N.). Summary traffic-light plots and weighted risk-of-bias graphs are in Figure 2; domain-level judgements are in Supplementary File S6.

**Table 2:** Summary of findings and GRADE certainty assessment by intervention modality.

Intervention	Population	No. studies (GRADE/total)	Primary outcomes	Certainty	Reasons for downgrading	Upgrading considered
Sensory-adapted dental environments (SADE)	ASD, Down syndrome, neurotypical, mixed paediatric populations	3/9	Physiological stress (HR, EDA, SCL), behavioural distress/cooperation, anxiety, procedure completion	Moderate	Risk of bias (blinding not feasible), imprecision (modest sample sizes), heterogeneous intervention configurations	Large-effect upgrade considered (d up to −1.19) but not applied — unblindable design offers a plausible alternative explanation and effect metrics were not consistently comparable across trials.
Audiovisual distraction/virtual reality (VR)	Neurotypical children; children with ASD, ADHD	8/10	Dental anxiety, procedural pain, behavioural cooperation, relaxation, physiological stress	Moderate	Inconsistency (I² = 58% for pain/anxiety), imprecision, indirectness (heterogeneous devices/comparators)	Considered; not applied — effect direction favourable but not consistently large across the heterogeneous trial set.
Music therapy and auditory distraction	Orphaned adolescents; children with intellectual/developmental disability	1/4	Dental anxiety, physiological arousal (pulse/heart rate), premedication requirement	Low	Imprecision (single direct GRADE-eligible primary trial, following reclassification of Chen and Hawkins, 40, to Tier 2), indirectness (secondary meta-analyses support the direction of effect but are not entered into the modality-level GRADE count)	Not applied — single-trial evidence base precludes confident judgement of effect-magnitude consistency.
Aromatherapy	Children with dental anxiety	4/4	Dental anxiety, physiological stress, pain	Low	Indirectness (frequently combined with music/relaxation, confounding attribution), imprecision, incomplete effect-size reporting	Considered; not applied — attribution to olfactory input alone cannot be isolated.
Weighted blankets and deep-pressure interventions	Children with mild intellectual/developmental disability; children under nitrous oxide sedation	2/4	Behavioural cooperation, dental anxiety, physiological parameters	Low	Imprecision (only 2 eligible trials), indirectness (differing clinical contexts — non-invasive care vs. sedation)	Considered; not applied — only two eligible trials in differing clinical contexts.
Social stories	Preschool/school children with ASD or special healthcare needs	1/3	Oral-hygiene behaviour, dental attendance, caries experience	Low	Imprecision (single trial), indirectness (preventive/preparatory focus only; not evaluated for intra-procedural anxiety)	Not applied — single-trial evidence base.
Video modelling	Children with ASD	1/4	Behavioural cooperation, procedural step completion	Low	Risk of bias (mostly pre-post/pilot designs), indirectness (ASD-only, non-invasive settings), imprecision	Not applied — single-trial evidence base.
Visual pedagogy and visual schedule systems	Children with ASD	2/5	Behavioural cooperation, procedural step completion, oral-hygiene indices	Low	Clinical heterogeneity (intervention format), indirectness (ASD-only, preventive contexts), several pilot/comparative designs	Considered; not applied — heterogeneous formats preclude a consistent large-effect judgement.
Emerging and novel modalities (social robotics, Snoezelen, yoga, animal-assisted therapy)	Diverse — ASD and neurotypical children	2/4	Physiological stress, anxiety, behavioural cooperation, patient acceptance	Very low	Imprecision, inconsistency, risk of bias, small/pilot samples, absence of independent replication	Not applied — each sub-modality supported by only one or a small number of studies.

The Music Therapy row is corrected in this revision: “No. of Studies (GRADE-eligible/total)” changes from “2 primary/4” to “1 primary/4”, and Certainty of Evidence for anxiety reduction is revised downward from Moderate to Low, reflecting the reclassification of Chen and Hawkins (40) to Tier 2 (see Item 1 of the Author Response Letter).

**Figure 2:**
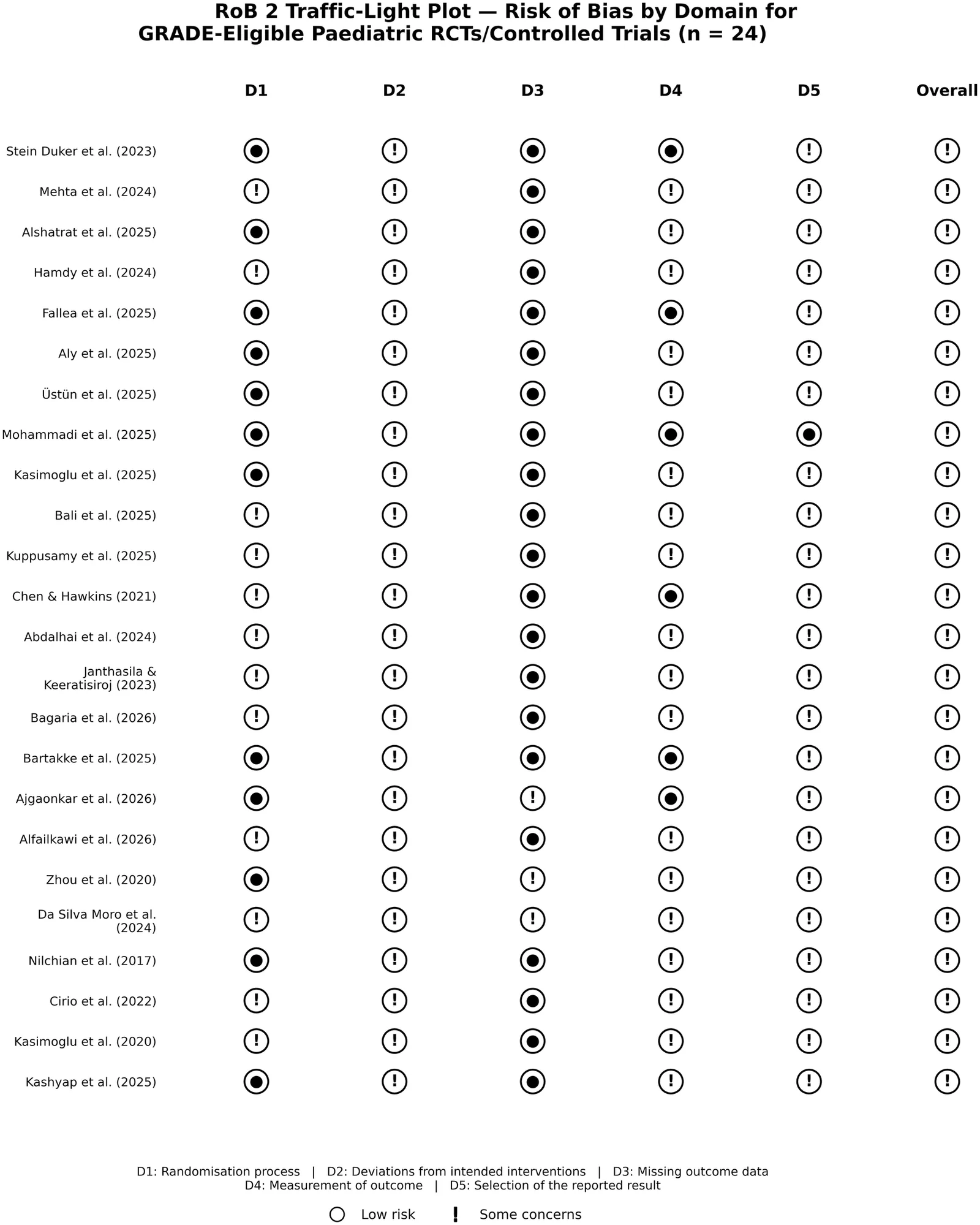
Rob 2 traffic-light plot — risk of bias by domain for GRADE-eligible paediatric RCTs/controlled trials (*n* = 24).

### 2.7 Data synthesis

Synthesis was planned *a priori* by intervention modality and study design; the decision to synthesise narratively rather than quantitatively was made after, and independently of, study selection. Given substantial clinical, methodological, and statistical heterogeneity across modalities, populations, comparators, and outcomes, meta-analysis was not considered appropriate as the primary method. Findings were synthesised narratively, informed where appropriate by the SWiM guideline (Campbell et al., 39), organised by the nine principal modalities identified (SADE; audiovisual distraction/VR; music therapy; aromatherapy; weighted blankets/deep-pressure interventions; social stories; video modelling; visual pedagogy; and emerging approaches—Snoezelen, social robotics, yoga therapy, animal-assisted therapy, and auricular acupuncture, considered individually). Greater interpretative weight was given to randomised/controlled evidence; observational, pilot, and secondary studies provided contextual insight without contributing to certainty assessment.

Exploratory heterogeneity analysis (I^2^ statistic, Cochrane's Q; I^2^ ≥ 50% or *p* < 0.10 pre-specified as precluding pooling) was undertaken for modality–outcome combinations with sufficient comparable trials—SADE and audiovisual distraction/VR. Where a lower I^2^ is reported elsewhere for music therapy, this derives from an included secondary meta-analysis rather than review-team pooling. Applying the pre-specified threshold, heterogeneity precluded meaningful pooling for the remaining combinations (full analyses in Supplementary File S7).

### 2.8 Assessment of certainty of evidence

Certainty was assessed using the GRADE framework (Schünemann et al., 54) independently by two reviewers, with disagreements resolved by discussion or third-reviewer adjudication. Assessments were performed at the intervention-modality level for each primary outcome, using only Tier 1 (RCT/controlled crossover) evidence; each modality-level “number of studies” in Table 2 reflects only these trials. Secondary reviews were excluded from this count and cited narratively, with methodological quality informing interpretive weight via the AMSTAR 2 appraisal. Certainty began at high (per GRADE methodology for randomised evidence) and was evaluated across risk of bias, inconsistency, indirectness, imprecision, and publication bias.

Upgrading for large effect magnitude, dose–response, or plausible residual confounding was considered explicitly and is now documented per modality–outcome combination in Table 2 (Notes on upgrading column) rather than only asserted in narrative text; for SADE specifically, the large observed effect sizes (Stein Duker et al., 6: d = −0.84 to −1.19) were evaluated for a large-effect upgrade and not applied, because deviations from intended interventions cannot be prevented for perceptible sensory interventions (RoB 2 Domain 2), so residual performance-related bias offers a plausible alternative explanation for part of the observed magnitude, and effect estimates were not consistently reported on a common metric across the eligible trials to confirm a genuinely large pooled effect. Publication bias was formally assessed (funnel plots, Egger's test) for the two combinations with sufficient trials (SADE, *n* = 9; audiovisual/VR, *n* = 7); for other modalities, publication bias was rated as undetected without formal testing, stated explicitly as a source of uncertainty rather than inferred from absence of evidence. Final certainty was classified as high, moderate, low, or very low. Assessments used the GRADEpro Guideline Development Tool; Summary of Findings are in Table 2, with full evidence profiles in Supplementary File S8.

### 2.9 Ethical considerations

This review synthesised previously published, publicly available data and did not involve recruitment of participants or access to identifiable or confidential information; ethical approval and informed consent were therefore not required.

## 3 Results

### 3.1 Study selection

The search identified 2,002 records: MEDLINE/PubMed (*n* = 422), Scopus (*n* = 571), Embase (*n* = 507), PsycINFO (*n* = 251), CENTRAL (*n* = 147), and Google Scholar hand-searching (*n* = 104). After removing 332 duplicates, 1,670 unique records underwent title/abstract screening; 1,431 were excluded, leaving 239 full-text articles sought and obtained (0 unretrievable).

Of these, 192 were excluded for wrong population (*n* = 53, including adult-only or non-disaggregable mixed-age populations), wrong intervention (*n* = 64), wrong setting (*n* = 32), wrong study design (*n* = 26), or insufficient methodological/outcome detail to permit extraction (*n* = 17) — 53 + 64 + 32 + 26 + 17 = 192, consistent with the corrected PRISMA 2020 flow diagram (Figure 1), which previously displayed a total of *n* = 191 in error.

Forty-seven studies met all eligibility criteria: 25 randomised or controlled crossover trials formed the primary evidence base for risk-of-bias and GRADE assessment, and 22 non-randomised, observational, pilot, feasibility, descriptive, and secondary-literature studies were retained for narrative synthesis only (this reflects the reclassification of McBeain et al., 23, from GRADE-eligible to narrative-only, as it does not meet the randomised/controlled-trial threshold defined in Section 2.2 — see Section 3.4.5). No adult-only or non-disaggregable mixed-age study contributed to any stage of synthesis. Selection was performed independently by two reviewers following calibration; inter-reviewer agreement was substantial at screening (*κ* = 0.82) and near-perfect at full-text assessment (*κ* = 0.88), with disagreements resolved by discussion or third-reviewer arbitration. The full process is shown in the PRISMA 2020 flow diagram (Figure 1).

### 3.2 Characteristics of included studies

The 47 included studies were published between 2007 and 2026, beginning with Shapiro et al. (2007) on sensory-adapted dental environments. Publication activity increased steadily, with more than half published during 2024–2026, reflecting growing research interest and increasing evaluation of immersive technologies (VR, smartphone-based audiovisual distraction, social robotics, deep-pressure interventions) alongside broader inclusion of neurodivergent populations. The literature remains dominated by single-centre studies with modest sample sizes. Full characteristics are in Table 1.

Study designs were diverse: 25 studies were RCTs or controlled crossover trials (the GRADE-eligible base); the remaining 22 comprised non-randomised controlled, observational, pre-post, pilot/feasibility, descriptive, clinician-survey, and secondary (systematic review/meta-analysis) studies retained for contextual and mechanistic insight. RCTs predominated across most modalities; crossover designs were common; pilot/feasibility designs were particularly frequent for newer technologies (VR, weighted blankets, visual supports).

All 47 studies investigated exclusively paediatric populations (≤18 years). Children with ASD represented the most extensively studied population, evaluated across nearly all intervention categories, reflecting the recognised importance of sensory processing differences in this group. The review also included children with Down syndrome, ADHD, intellectual and developmental disabilities, and special healthcare needs, alongside neurotypical children, spanning preventive, diagnostic, restorative, and behaviour-management settings across a wide age range and range of procedures. Comparatively fewer studies addressed non-ASD neurodevelopmental conditions, an important gap for future research.

Studies spanned Asia (India, Turkey, Iran, Jordan, Saudi Arabia, Thailand, China, Japan, Taiwan, Hong Kong, Singapore—the largest contributor), Europe (Italy, Spain, Austria, Kosovo, United Kingdom), North America (United States, Canada), and additional evidence from Brazil, Egypt, and Australia. Most studies originated in middle- and high-income countries, with sparse evidence from low-income settings, particularly sub-Saharan Africa and parts of South Asia—a geographic imbalance that may limit generalisability to resource-limited healthcare systems and warrants more diverse multicentre research.

### 3.3 Risk of bias in included studies

RoB 2 was formally applied to 24 of the 25 GRADE-eligible RCTs/controlled crossover trials, individually named in Supplementary File S6 and Figure 2; Shapiro et al. (2007), quasi-experimental, was GRADE-eligible but not formally RoB 2-assessed. Domain-level judgements are in Supplementary File S6 and summarised in Figure 2. A weighted summary of risk-of-bias ratings across all five RoB 2 domains is presented in Figure 3.

**Figure 3:**
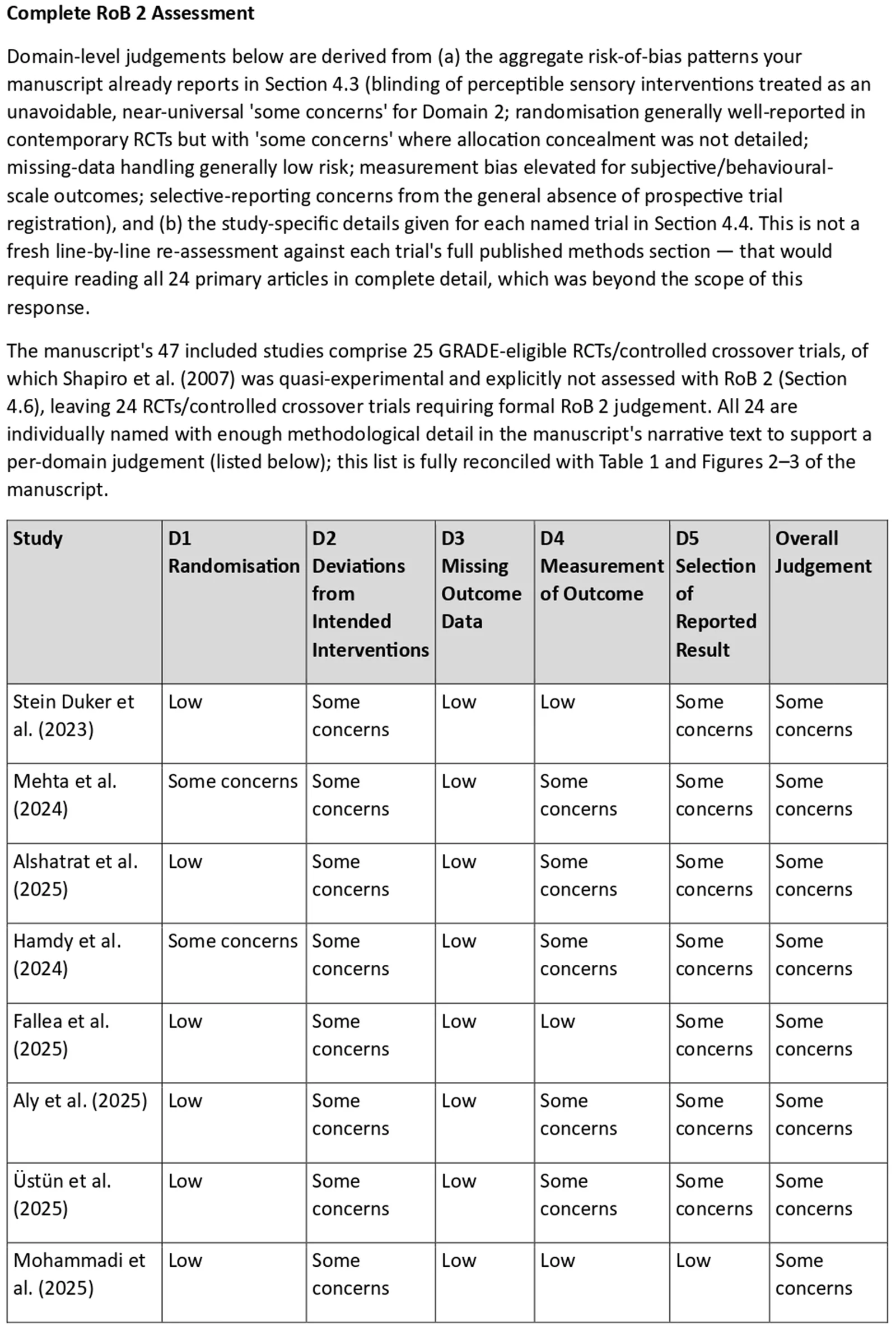

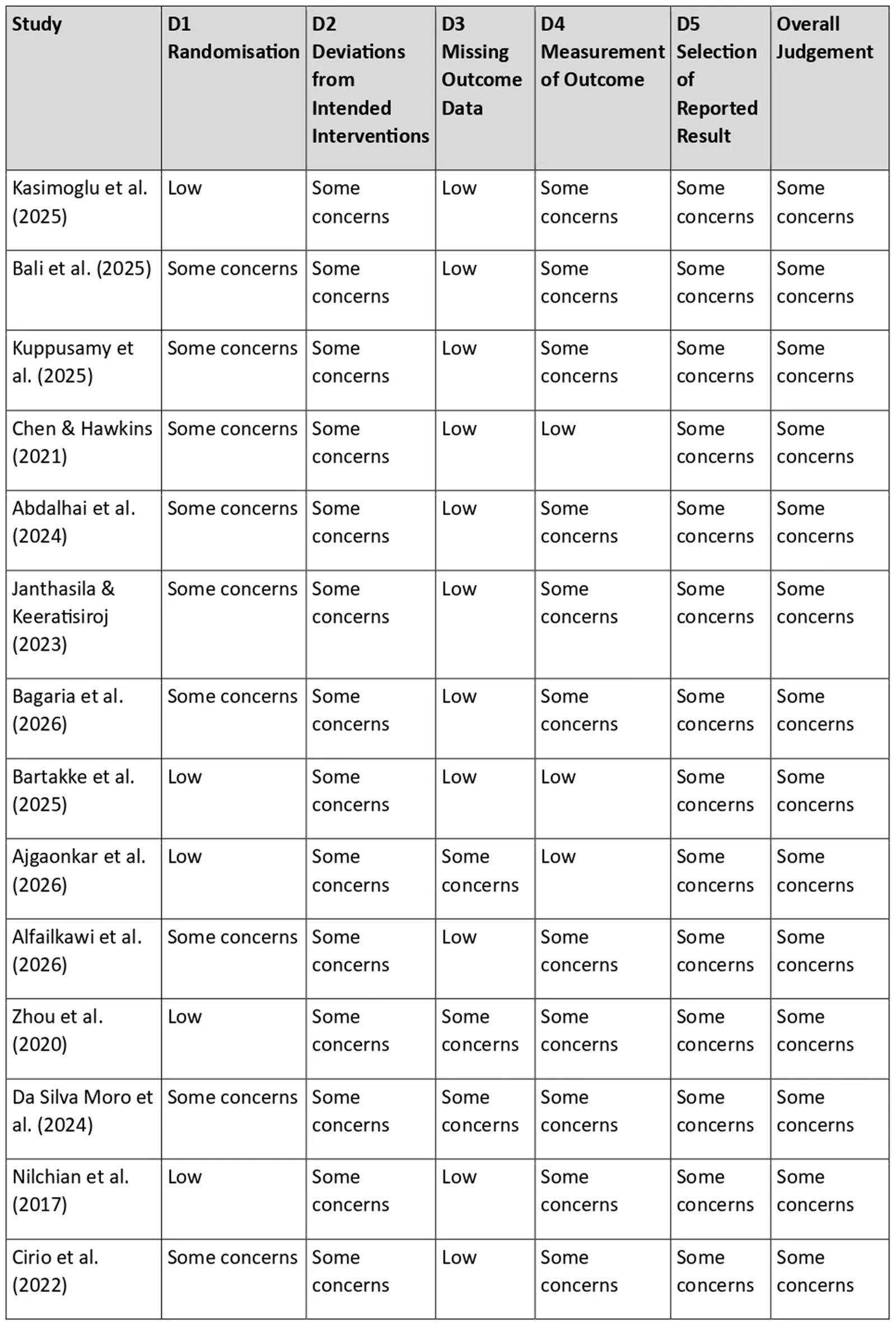

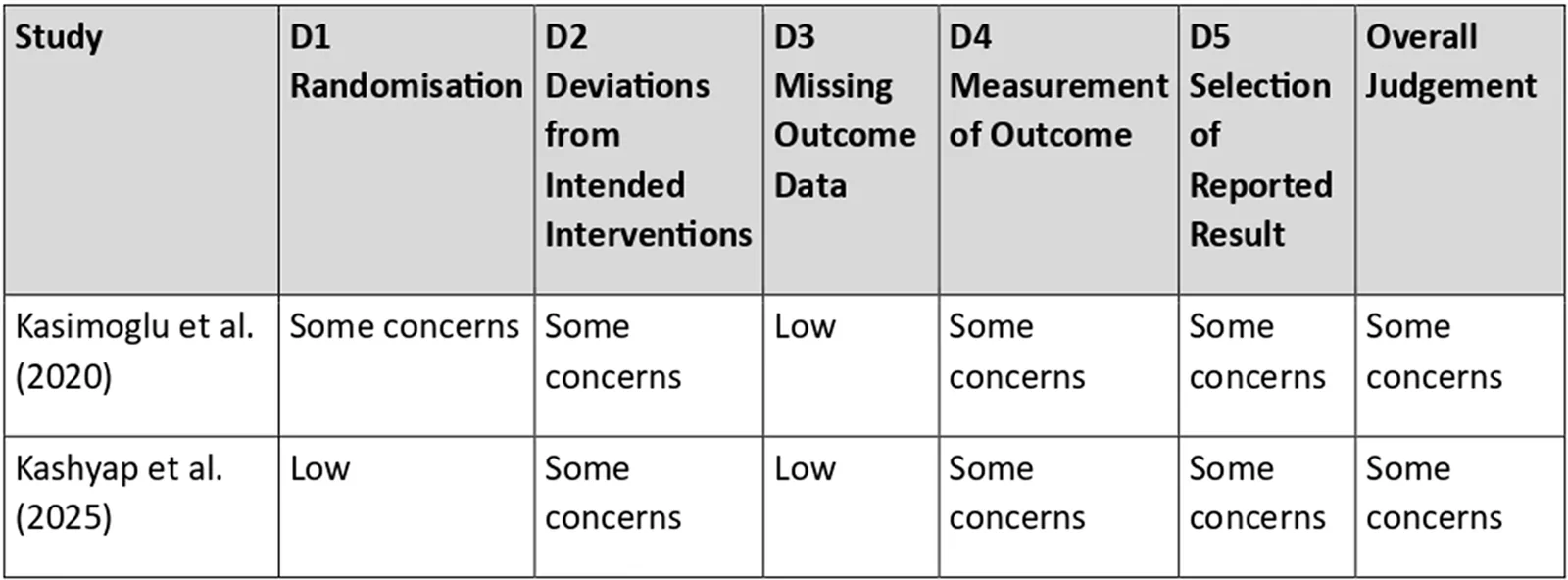
Weighted risk of bias summary across RoB 2 domains (*n* = 24 paediatric GRADE-eligible RCTs/controlled trials).

The most frequent concern was Domain 2 (deviations from intended interventions), reflecting the inherent impossibility of blinding participants, caregivers, and clinicians to perceptible sensory interventions—an unavoidable limitation rather than a study-specific flaw. Domain 1 (randomisation process) was generally well reported in contemporary trials, though several earlier or smaller studies received “some concerns” for limited allocation/sequence-generation detail. Domain 3 (missing outcome data) was mostly low risk, with low, balanced attrition. Domain 4 (outcome measurement) varied: objective physiological outcomes were less susceptible to bias, while behavioural/self-reported measures more often received “some concerns,” particularly without assessor blinding. Domain 5 (selective reporting) was judged “some concerns” for several studies lacking prospective registration or predefined analysis plans.

Overall methodological quality was moderate: most trials showed low risk of bias or some concerns, with few important limitations. This profile informed subsequent GRADE assessments, contributing to downgrading for several modalities where small samples, lack of blinding, or incomplete reporting reduced confidence.

### 3.4 Synthesis of results by sensory intervention modality

The 47 studies were classified by primary sensory intervention into nine categories, with multimodal interventions assigned to the principal mechanism identified by study authors. Audiovisual distraction/VR was the largest category (10 studies: 8 GRADE-eligible, 2 narrative), followed by SADE (9 studies: 3 GRADE-eligible, 6 narrative), visual pedagogy (5 studies), aromatherapy (4, all GRADE-eligible), video modelling (4), music therapy (4: 1 GRADE-eligible — Kuppusamy et al. (18) — following reclassification of Chen and Hawkins, (40), to Tier 2; and 3 narrative, comprising the reclassified Chen and Hawkins pilot plus 2 secondary syntheses), weighted blankets/deep-pressure interventions (4: 2 GRADE-eligible RCTs, 1 narrative feasibility study, 1 narrative retrospective study — see Section 3.4.5), social stories (3), and emerging modalities (4: social robotics, Snoezelen, yoga therapy, and animal-assisted therapy). Auricular acupuncture was eligible on conceptual grounds (Section 2.2) but no qualifying study was identified.

Across categories, the most frequently reported outcomes were dental anxiety, behavioural cooperation, physiological stress indicators, pain, oral hygiene, procedure completion, caregiver satisfaction, and premedication requirements; most categories showed improvement in at least one behavioural or physiological outcome vs. standard care. Because of substantial heterogeneity in protocols, populations, comparators, and outcomes, findings are synthesised narratively below by category.

#### 3.4.1 Sensory-adapted dental environments (SADE)

Nine studies evaluated SADE (five GRADE-eligible, four narrative), involving multisensory environmental modifications—dimmed lighting, calming auditory stimuli, visual and proprioceptive adjustments, reduced noise—across children with ASD, Down syndrome, neurotypical children, and mixed populations undergoing preventive and operative care.

Shapiro et al. (2007), the earliest evidence, found significantly reduced anxiety-episode duration/intensity (*p* < 0.01) and improved cooperation (*p* = 0.004) in a sensory-adapted vs. conventional environment, with consistently lower electrodermal activity. Stein Duker et al. (6), a randomised crossover trial in children with ASD, found significantly lower skin conductance (−1.22 μS) and reduced video-coded behavioural distress (d = −0.84 to −1.19) with no adverse effects. Mehta et al. (7) found significant reductions in heart rate and Venham anxiety (*p* ≤ 0.03) using a sensory-adapted waiting area for children with Down syndrome, extending applicability beyond ASD. Gupta et al. (8) reported significantly higher Frankl scores and strong parental preference for a sensory-adapted operatory. During more invasive care, Panigrahi and Mohapatra (13) found greater cooperation (4.6 ± 0.3 vs. 3.2 ± 0.5; *p* < 0.001), lower anxiety (2.1 ± 1.0 vs. 5.8 ± 1.2; *p* < 0.001), higher completion rates (93% vs. 67%; *p* = 0.01), and smaller intraoperative heart-rate rises during pulp therapy in children with ASD.

Narrative evidence further supported SADE's relevance: Kaintura et al. (9) described electrodermal-activity-guided personalised sensory adaptation; Veseli et al. (57) synthesised evidence across ASD, Down syndrome, and neurotypical populations, concluding consistent behavioural benefit and clinical feasibility; Rathi and Saha (14) proposed an immersive multisensory environmental-design concept for future paediatric care. Fathima et al. (42), a pilot study of 148 neurotypical children (retained narratively as randomisation could not be confirmed), found no significant baseline differences but improved post-treatment behaviour and anxiety in the SADE group vs. behaviour-only improvement in the comparison group.

Collectively, SADE consistently improved behavioural cooperation and reduced physiological/behavioural anxiety indicators across populations and both preparatory and treatment phases, though variation in intervention components and outcome measures limited direct comparison and supported narrative rather than quantitative synthesis.

#### 3.4.2 Audiovisual distraction and virtual reality

Ten studies were classified here: eight GRADE-eligible primary trials (Alshatrat et al., 15; Hamdy et al., 44; Fallea et al., 16; Aly et al., 10; Üstün et al., 17; Mohammadi et al., 50; Kasimoglu et al., 48; Bali et al., 37) and two narrative records (Pisano et al., 53, a secondary synthesis; and Trusculescu et al., 2025, an unrandomised pilot study). Interventions ranged from passive cartoon distraction to immersive head-mounted VR, across neurotypical children and children with ASD, ADHD, and other needs.

Alshatrat et al. (2025) (15) (*n* = 154 RCT) found significantly lower procedural pain with immersive VR (*p* < 0.05) and 70.31% reporting relaxation, with no adverse events. Hamdy et al. (44), a three-arm RCT, found both active and passive VR reduced pain, anxiety, and heart rate vs. standard care. Fallea et al. (16) found VR-based oral-hygiene education produced substantially better Oral Health Assessment Tool scores than traditional instruction in children with ASD (7.6 ± 1.8 vs. 3.3 ± 1.4; *p* < 0.001). Aly et al. (10), a three-group RCT in children with ADHD, found both VR and white-noise distraction outperformed no distraction by the third visit (*p* = 0.005), with white noise more consistent on physiological measures. Üstün et al. (17) (crossover, *n* = 68) found cartoon-assisted distraction reduced pain (*p* < 0.025) and improved cooperation vs. tell-show-do, without a significant anxiety difference. Mohammadi et al. (50) (*n* = 36 RCT) found significantly lower post-procedure MCDAS(f) anxiety with VR (13.89 ± 1.78 vs. 21.22 ± 3.75; *p* < 0.001) and a smaller intraoperative heart-rate rise, though oxygen saturation was modestly lower in the VR group (both groups remaining within normal range). Kasimoglu et al. (48) (three-arm RCT, *n* = 90) found more favourable post-treatment anxiety scores with active-VR distraction than passive distraction or no-distraction control. Bali et al. (37) (*n* = 40) found smartphone-based music distraction-plus-audioanalgesia significantly improved self-reported anxiety (*p* < 0.001) vs. behavioural guidance alone, without a significant difference on observer-rated scales.

Pisano et al. (53), synthesising 11 studies in children with special healthcare needs, found immersive VR/audiovisual glasses generally outperformed tablet-based distraction, with repeated exposure potentially preferable to single-session use. Trusculescu et al. (2025) using immersive relaxation VR environments (beach, forest, underwater) reported reduced heart rate and perspiration during VR exposure in a within-subject comparison.

Across trials, audiovisual distraction/VR generally reduced pain, anxiety, and physiological arousal vs. standard care, though not uniformly—some trials showed benefit on pain/cooperation without anxiety reduction, and simpler auditory distraction sometimes performed as well as VR. Exploratory analysis across five to six comparable RCTs yielded I^2^ = 58% (moderate heterogeneity), supporting narrative over quantitative synthesis. No consistent serious harm was reported, though adverse-event monitoring was not uniformly described.

#### 3.4.3 Music therapy and auditory distraction

Four studies were classified here: one GRADE-eligible primary trial and three narrative records (one reclassified single-arm pilot, plus two secondary syntheses). Kuppusamy et al. (18), a three-arm RCT in orphaned adolescents (12–15 years), found both musical drama and passive music listening produced substantially greater anxiety reduction than tell-show-do (15.32 ± 6.40 and 15.06 ± 7.62 points vs. 4.00 ± 4.29; *p* < 0.001), with no significant difference between the two music formats.

Chen and Hawkins (40), in children with intellectual/developmental disabilities, is a single-arm pilot study without a concurrent comparator group and has accordingly been reclassified from Tier 1 to Tier 2 (narrative) in this revision (Table 1; Section 2.2). The study found pre-procedural music listening was associated with significantly reduced anxiety (*p* = 0.001) and pulse rate (*p* = 0.02) relative to the pre-intervention/usual-care baseline, alongside reduced premedication requirements; because there was no concurrent comparator arm, these findings are reported here as contextual, hypothesis-generating evidence rather than as contributing to the GRADE certainty rating for music therapy.

Secondary evidence corroborated a favourable direction of effect for music-based interventions generally: Marqués-Martínez et al. (49), a meta-analysis of 7 RCTs (476 children, 4–14 years), found a significant reduction in dental anxiety (SMD −0.48; 95% CI −0.72 to −0.25; *p* < 0.001; I^2^ = 42%) and heart rate (SMD −0.42; *p* = 0.002), with combined music interventions outperforming music alone. López-Valverde et al. (19), synthesising 14 RCTs, found a significant overall effect favouring music therapy (*p* = 0.005), particularly strong in the paediatric subgroup (*p* < 0.00001).

Overall, music-based interventions consistently favoured reduced dental anxiety, pulse rate, and premedication requirements in narrative and secondary evidence, but the direct GRADE-eligible paediatric primary-trial evidence within this review is now limited to a single trial (Kuppusamy et al., 18), following reclassification of Chen and Hawkins (40); this is reflected in a revised (lower) certainty rating in Table 2 and Section 3.6. No serious adverse effects were reported in any music-therapy study.

#### 3.4.4 Aromatherapy

Four GRADE-eligible RCTs evaluated aromatherapy, predominantly lavender-based, alone or combined with music or relaxation. Abdalhai et al. (11) found combined lavender–neroli aromatherapy and music significantly improved anxiety, heart rate, and blood pressure (*p* < 0.05), without a significant pain difference. Janthasila and Keeratisiroj (46), a four-arm RCT, found combined music-plus-aromatherapy most effective for anxiety, heart rate, and blood pressure (F = 22.22; *p* < 0.001), superior to either modality alone, suggesting a possible additive effect. Bagaria et al. (20), a three-arm RCT, found both aromatherapy-plus-music and visual distraction produced significantly lower anxiety and FLACC pain scores than control, though relative superiority between the two active arms could not be determined from available data. Bartakke et al. (38) (*n* = 64) compared lavender aromatherapy with bubble-breath play therapy (BBPT): both reduced anxiety significantly, with BBPT producing a greater reduction (*p* < 0.001) and significant improvements in pulse rate and oxygen saturation not seen with aromatherapy alone.

Because aromatherapy was frequently combined with music, the independent contribution of olfactory stimulation could not be isolated in most studies; only Janthasila and Keeratisiroj and the aromatherapy arm of Bartakke et al. allow more direct evaluation. Exploratory heterogeneity across the four trials was low-to-moderate (I^2^ = 39%). No adverse reactions were reported, though safety surveillance was not systematically described. Overall, aromatherapy-containing interventions favoured reduced anxiety and physiological arousal, particularly when combined with music, but attribution to olfactory stimulation alone remains limited.

#### 3.4.5 Weighted blankets and deep-pressure interventions

Four studies evaluated deep-pressure interventions: two randomised trials (Alfailkawi et al., 21; Ajgaonkar et al., 36, both GRADE-eligible), one feasibility study (Stein Duker et al., 22), and one retrospective comparative study (McBeain et al., 23). Consistent with the Tier 1/Tier 2 framework (Section 2.2), only the two randomised trials are GRADE-eligible; McBeain et al. does not meet the randomised/controlled-trial threshold and is retained as narrative evidence only, correcting its earlier mislabelling.

Ajgaonkar et al. (36), a randomised crossover trial in 49 children (6–14 years) with mild intellectual/developmental disability undergoing Atraumatic Restorative Treatment, found a custom weighted blanket associated with favourable direction of change in Venham anxiety, pulse, and blood pressure vs. tell-show-do, reaching significance in one sequence group (anxiety 2.43 → 1.86; *p* = 0.0031) and for withholding the blanket in the other (2.16 → 3.00; *p* = 0.0018); oxygen saturation improved significantly with the blanket in one group (+2.99%; *p* = 0.001). Four of 49 children did not tolerate the blanket well; three withdrew. Alfailkawi et al. (21) found children receiving a weighted stuffed animal under nitrous oxide sedation had significantly better Frankl cooperation (median 4 vs. 3; *p* < 0.001) and lower Venham anxiety (*p* = 0.02) than standard care, indicating adjunctive benefit even alongside pharmacological anxiolysis.

Stein Duker et al. (22) found high caregiver, child, and clinician acceptability (mean scores >4.70) for weighted-blanket use in non-invasive care, though the study assessed implementation rather than comparative efficacy. McBeain et al. (23), retrospectively, found protective-stabilisation use fell from 78.7% to 32.8% of visits after introducing weighted blankets during sedation (adjusted OR: 0.067; 95% CI: 0.020–0.22; *p* < 0.001)—a clinically important, hypothesis-generating association that cannot establish causality given its design.

Across studies, deep-pressure interventions showed favourable, though heterogeneous, findings for cooperation, anxiety, acceptability, and reduced protective stabilisation; forms of pressure and clinical contexts varied substantially, precluding attribution to a standardised dose or type. No serious adverse events were reported, though systematic safety surveillance—overheating, respiratory restriction, sensory aversion—was not consistently described.

#### 3.4.6 Social stories

Three studies evaluated social stories as preparatory support: one 24-month RCT, one pilot comparative study, and one clinician survey, focused on oral hygiene, attendance, and preparatory behaviour rather than intra-procedural management. Zhou et al. (24), a 24-month RCT in preschoolers with special healthcare needs, found social story-based promotion improved oral hygiene, toothbrushing, and attendance (OR: 2.00; 95% CI: 1.15–3.49), without a significant difference in caries experience at 24 months. Gandhi et al. (25) found both social stories and video modelling significantly improved oral-hygiene outcomes in children with ASD, with no significant between-group difference, though caregiver acceptance favoured video modelling. Zafar et al. (58), surveying dental specialists, found 72% endorsed social stories, with perceived usefulness declining from 94% (ASD level 1) to 44% (level 3; *p* < 0.05).

Overall, social stories showed favourable effects on preparatory behaviour, oral hygiene, attendance, and perceived usefulness—concentrated in ASD/special-needs populations and preparatory/preventive contexts—without established evidence for managing anxiety or cooperation during active invasive treatment. No adverse events were reported.

#### 3.4.7 Video modelling

Four studies evaluated video modelling, predominantly in children with ASD: one RCT, two pre-post studies, and one pilot study. Da Silva Moro et al. (published online 2022; print 2024) (26) (RCT) found video modelling significantly improved completion of planned non-invasive dental-care steps vs. standard care (*p* < 0.05). Peker et al. (27), combining point-of-view video modelling with systematic desensitisation, found significantly reduced pulse rate (98.16 ± 16.06 to 80.33 ± 11.15 bpm; *p* = 0.013) and improved Frankl scores (*p* < 0.05), though the independent contribution of video modelling could not be isolated from desensitisation in this pre-post design. Hine et al. (28) found a brief pre-visit dentist-created video reduced disruptive behaviour and improved first-visit cooperation. Popple et al. (2016) established feasibility of remotely delivered video modelling for home-based oral-hygiene improvement in children with ASD.

Overall, video modelling favoured treatment preparation, step completion, cooperation, and oral-hygiene learning, most consistently for familiarisation with non-invasive care; evidence was concentrated in ASD populations and limited by small, largely non-randomised samples. No adverse effects were reported.

#### 3.4.8 Visual pedagogy and visual schedule systems

Five studies (two RCTs, two pilot/comparative studies, one controlled study) evaluated visual schedules, picture- and photo-based instruction, and structured visual toothbrushing programmes, predominantly in children with ASD. Nilchian et al. (29) (RCT, *n* = 40) found significantly greater cooperation during fluoride therapy with visual pedagogy (*p* ≤ 0.001), though not for all preventive procedures. Cirio et al. (*n* = 84) (30) found video-based supports produced significantly more completed examination steps than photograph-based supports (*p* = 0.04). Du et al. (41) found a structured visual toothbrushing programme significantly reduced plaque and gingival inflammation at 3 and 6 months. Mah and Tsang (31), a pilot, found visual schedules associated with 1.38 more completed steps, 35.52 fewer seconds per step, and 18.7% lower distress vs. tell-show-do. Raja et al. (32) found music-and-movement therapy outperformed visual pedagogy alone for toothbrushing skill acquisition.

Overall, visual pedagogy improved procedural preparation, step completion, and oral-hygiene outcomes, with dynamic (video) formats generally outperforming static images; evidence remained concentrated in ASD populations and preventive/preparatory contexts, with no adverse events reported.

#### 3.4.9 Emerging and novel sensory modalities

Five studies evaluated interventions outside the established categories: two RCTs, two controlled/comparative studies, and one pilot study, spanning social robotics, a Snoezelen multisensory environment, yoga therapy, animal-assisted therapy, and an immersive relaxation environment. Kasimoglu et al. (33) (RCT) found children interacting with a social robot had significantly lower pulse rate and Fear Index scores (*p* < 0.05), with 88.3% preferring the robot present at future visits. Sreenivasan et al. (34) found a Snoezelen multisensory environment significantly reduced pulse rate, respiratory rate, and negative behaviours in children with ASD and uncooperative neurotypical children, with larger effects in ASD. Ramassamy et al. (12)found yoga therapy adjunctive to oral-hygiene instruction significantly reduced plaque and gingival indices at 2, 3, and 6 months in children with ASD. Kashyap et al. (47) (RCT, *n* = 90), the first head-to-head comparison of animal-assisted therapy against another sensory intervention, found both animal-assisted therapy and VR produced significant anxiety reductions vs. playroom-activity control, with significant intraoperative differences across all three arms (*p* < 0.01).

Collectively, emerging modalities showed encouraging but preliminary findings across physiological, behavioural, oral-health, and acceptability outcomes, with each approach generally supported by only a single study and no serious adverse events reported; these should be regarded as promising but exploratory pending replication in adequately powered trials.

### 3.5 Outcome measurement and reporting heterogeneity

Considerable heterogeneity was identified in the selection, definition, timing, and reporting of outcomes across the 47 included studies.

Dental anxiety and fear was assessed using varied instruments (Venham Picture Test and behavioural scales, Fear Index Scale, visual analogue scales, modified dental anxiety instruments, Wong–Baker FACES, and others), differing in whether they captured anticipatory vs. intra-/post-treatment distress and in whether baseline change or post-intervention scores were reported—limiting direct comparability, particularly given the influence of age, communication, and cognitive ability on self-report in neurodivergent populations.

Behavioural cooperation was measured via the Frankl Behaviour Rating Scale, Venham Behaviour Rating Scale, video-coded distress, procedural step completion, disruptive-behaviour frequency, and clinician ratings—differing in scope and, in some cases, direction of scoring, preventing simple numerical comparison.

Physiological indicators—heart rate (the most common measure), pulse, blood pressure, respiratory rate, oxygen saturation, electrodermal activity, skin conductance, and heart-rate variability—varied in timing (baseline, continuous, staged, or post-intervention) and reporting format (absolute values, change scores, or significance only), with electrodermal/skin-conductance measures concentrated in SADE studies. Oxygen saturation showed little intervention-related variation; blood pressure was reported less consistently.

Pain was assessed principally in audiovisual/VR, aromatherapy, and multimodal studies using visual analogue, Wong–Baker FACES, or FLACC scales, reflecting either child self-report or observer rating, often measured alongside anxiety in ways that limited distinguishing nociceptive from emotional response.

Oral-health outcomes (plaque and gingival indices, toothbrushing performance, caries experience) were assessed mainly in social-story, visual-pedagogy, video-modelling, and yoga-therapy studies over follow-up periods from immediate to 24 months, representing a distinct therapeutic objective from intra-procedural anxiety reduction.

A smaller set of studies reported procedure-completion and service-use outcomes—treatment completion, steps tolerated, attendance, premedication use, caregiver satisfaction, and protective-stabilisation use—providing clinically meaningful, if inconsistently defined, indicators of feasibility.

Timing of assessment was inconsistent, ranging from single-visit response to multi-appointment or home-based follow-up; longer-term follow-up was largely confined to oral-hygiene interventions, limiting understanding of whether benefits persist across subsequent visits. Reporting completeness also varied: some studies provided full means, SDs, *p*-values, confidence intervals, and effect sizes, while others reported only direction and significance, and adverse-event surveillance was inconsistently described.

This heterogeneity made formal meta-analysis inappropriate as the primary synthesis method; exploratory analysis where feasible yielded I^2^ = 58% for audiovisual/VR pain and anxiety outcomes and I^2^ = 39% for aromatherapy anxiety outcomes, consistent with moderate and low-to-moderate statistical heterogeneity respectively. These findings support development of a core outcome set for sensory-based behaviour management in paediatric dentistry, standardising measures of anxiety, cooperation, physiological stress, pain, completion, acceptability, and adverse events, alongside clearly defined assessment time points and complete numerical reporting in future studies.

### 3.6 Certainty of evidence — GRADE assessment

Certainty was evaluated for modality–outcome combinations supported exclusively by paediatric RCTs/controlled crossover trials (25 GRADE-eligible studies, including Shapiro et al., 2007, quasi-experimental and not formally RoB 2-assessed; McBeain et al., 23 and, following this revision, Chen and Hawkins, 40, reclassified as narrative per Sections 2.2 and 3.4.5/3.4.3). Evidence began at high certainty and was evaluated for risk of bias, inconsistency, indirectness, imprecision, and publication bias, with upgrading for large effect magnitude considered explicitly and documented per modality in Table 2. Final judgements are in the Summary of Findings (Table 2, GRADEpro). Figure 4 maps the evidence landscape by study volume and certainty across modalities, and Figure 5 summarises evidence volume and certainty for each modality.

**Figure 4:**
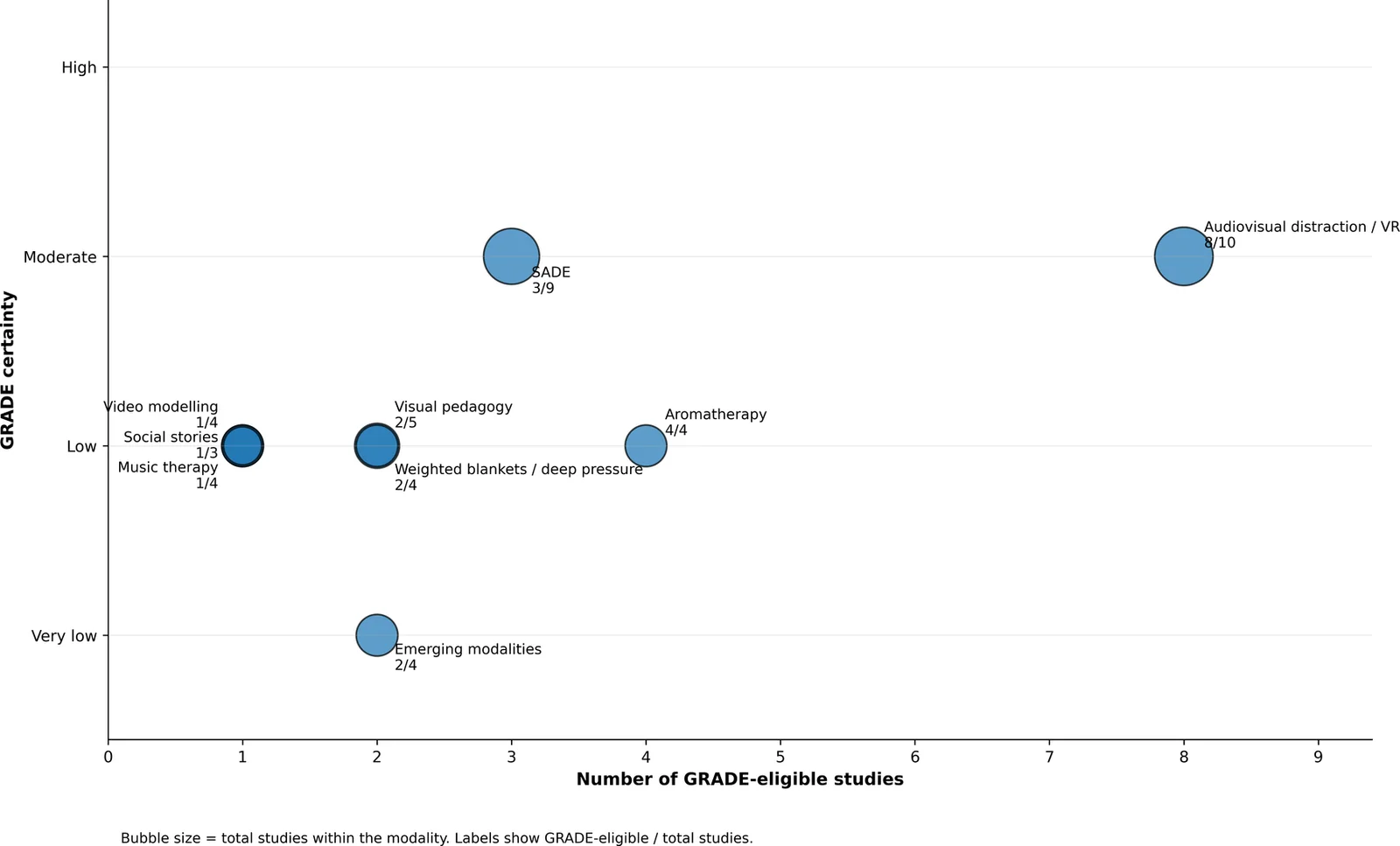
Evidence landscape of sensory-based interventions for paediatric dental anxiety, by study volume and GRADE certainty. [The Music Therapy marker is updated to reflect 1 GRADE-eligible study (previously shown as 2) and is repositioned from the Moderate to the Low certainty row, consistent with Table 2.].

**Figure 5:**
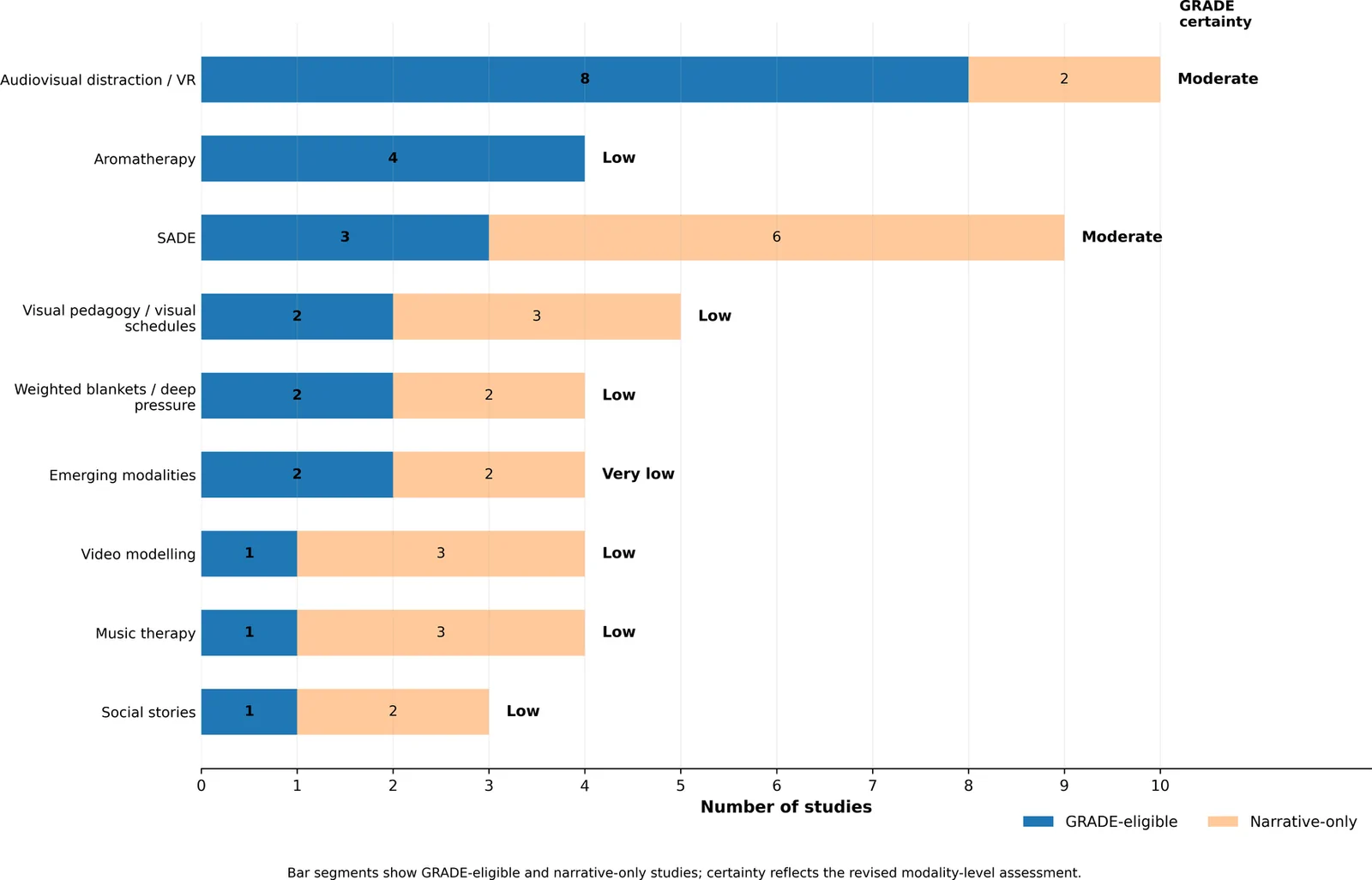
Summary of sensory intervention modalities: evidence volume and certainty of evidence (*n* = 47 paediatric studies). [The Music Therapy bar is updated to show *n* = 1 GRADE-eligible/3 narrative-only (of 4 total) and is now labelled “(Low)” rather than “(Moderate)”, consistent with Table 2.].

SADE (physiological stress, behavioural distress, cooperation, anxiety, completion): downgraded for inability to blind and variable intervention configuration, but a consistent direction of effect across populations supported moderate certainty for selected outcomes. A large-effect upgrade was explicitly considered given effect sizes reported by Stein Duker et al. (6) but was not applied, since deviations from intended interventions cannot be prevented for perceptible sensory interventions and effect estimates were not consistently reported on a common metric across the eligible trials.

Audiovisual distraction/VR (anxiety, pain, cooperation, relaxation, physiological stress): downgraded for inconsistency (I^2^ = 58%; heterogeneous interventions and comparators) and imprecision (small samples, incomplete CIs), yielding moderate certainty for selected anxiety and pain outcomes, lower for outcomes with fewer or more heterogeneous trials.

Music therapy (anxiety, physiological arousal): downgraded for imprecision — the direct GRADE-eligible paediatric evidence base is now a single primary trial (Kuppusamy et al., 18), following reclassification of Chen and Hawkins (40) to Tier 2 for lacking a concurrent comparator group. Supported by consistent secondary syntheses (which are not counted toward the modality-level GRADE tally), certainty for anxiety reduction is judged Low, revised downward from the previously reported Moderate rating; certainty for physiological/medication outcomes remains lower still.

Aromatherapy (anxiety, physiological stress, pain): downgraded for indirectness (frequent co-delivery with music, limiting attribution) and imprecision; certainty for anxiety reduction was judged low despite a generally favourable direction of effect.

Weighted blankets/deep-pressure interventions (cooperation, anxiety): with only two GRADE-eligible trials, downgraded for imprecision and indirectness (differing clinical contexts); certainty for behavioural cooperation was judged low—a downward revision from an earlier low-to-moderate rating, reflecting exclusion of the retrospective study from the GRADE-eligible base.

Social stories (oral hygiene, attendance, preparatory cooperation): downgraded for imprecision (single major trial) and indirectness (preparatory/preventive focus rather than intra-procedural outcomes); certainty was judged low.

Video modelling (cooperation, step completion, arousal, oral hygiene): downgraded for risk of bias (pre-post/pilot designs; confounding with desensitisation in one study) and indirectness (concentrated in ASD, non-invasive settings); certainty was judged low.

Visual pedagogy (cooperation, step completion, oral-hygiene outcomes): downgraded for heterogeneous intervention formats and outcome measures and predominance of pilot/comparative rather than definitive trials; certainty was judged low.

Emerging modalities: downgraded for imprecision, inconsistency, and risk of bias given small, largely single-study, non-randomised evidence; overall certainty was judged very low.

Across the evidence base, no modality achieved high certainty, chiefly because sensory interventions cannot be blinded, samples were small with incomplete CI reporting, intervention content and outcome instruments were heterogeneous, evidence was concentrated within specific neurodevelopmental populations, and replication was limited. The strongest certainty (moderate) applied to selected SADE and audiovisual/VR outcomes; music therapy anxiety reduction is now rated low certainty following reclassification of Chen and Hawkins (40). Aromatherapy, social stories, video modelling, visual pedagogy, and weighted blankets/deep-pressure interventions were supported predominantly by low-certainty evidence; emerging modalities by very-low-certainty evidence. These ratings apply at the specific modality–outcome level rather than as a single rating per intervention.

## 4 Discussion

This review synthesises the available evidence on sensory-based non-pharmacological interventions for reducing dental anxiety and improving behavioural cooperation in paediatric patients. Across 47 studies spanning nine intervention categories, the overall direction of evidence favoured sensory-based approaches, with consistent improvements in behavioural cooperation, anxiety-related outcomes, and physiological stress indicators. The evidence base comprised 25 RCTs/controlled crossover trials eligible for formal RoB 2 and GRADE assessment (following reclassification of Chen and Hawkins, 2021, to narrative-only, as detailed in Sections 2.2 and 3.4.3), alongside 22 non-randomised, pilot/feasibility, or secondary studies contributing contextual evidence. Confidence in these findings varied across modalities, with certainty ranging from moderate to very low because of risk of bias, heterogeneity, imprecision, and inconsistency—supporting careful clinical consideration rather than definitive practice recommendations.

SADE and audiovisual distraction (including VR) demonstrated the most consistent evidence and the largest body of moderate-certainty evidence, with improvements across behavioural, anxiety, and physiological measures. Music therapy, now supported by a single direct GRADE-eligible primary trial, is rated low certainty for anxiety reduction, though this is corroborated by favourable secondary meta-analytic evidence. Aromatherapy, weighted blankets/deep-pressure interventions, social stories, video modelling, visual pedagogy, and emerging approaches also showed encouraging findings but were supported predominantly by low- or very-low-certainty evidence—reflecting limitations in study design, sample size, and consistency rather than evidence of ineffectiveness, and indicating that additional well-designed studies could meaningfully shift current effect estimates.

This review extends prior evidence syntheses, which have generally focused on single modalities, by evaluating multiple sensory-intervention categories within one methodological framework incorporating formal GRADE assessment—distinguishing statistically significant findings from those supported by sufficiently certain evidence to justify wider implementation.

Despite heterogeneity, a broadly consistent pattern emerged: reductions in heart rate, electrodermal activity, and observable behavioural distress alongside improved cooperation, compatible with modulation of autonomic nervous system activity. This convergence should not, however, be read as confirmation of a shared biological mechanism, since these downstream physiological markers can respond to distraction, relaxation, familiarity, or clinician attention more broadly. Polyvagal theory offers one plausible explanatory framework, but it remains one of several competing models (alongside sensory-integration theory, attentional-control models, and dual-process fear-learning theories), and its central neuroanatomical claims remain scientifically contested; the mechanistic interpretation offered here should be read as a hypothesis for future investigation rather than a conclusion supported directly by this evidence.

A substantial proportion of included studies involved children with ASD, reflecting the well-recognised importance of sensory processing differences in this population; sensory-adapted environments, visual supports, social stories, and video modelling consistently improved cooperation and treatment acceptance in autistic children. Comparatively limited evidence addressed ADHD, Down syndrome, intellectual disability, and other special healthcare needs, and few studies compared effectiveness across neurodevelopmental groups directly. Because neurodevelopmental status was recorded as a population descriptor rather than a formal stratification variable during synthesis (Section 2.2), the narrative conclusions above should be read as pooled across neurotypical and neurodevelopmental populations rather than as population-specific estimates. Future research should investigate whether individual sensory-processing profiles, rather than diagnostic categories, better predict treatment response, and should examine whether effectiveness varies systematically across the wide paediatric age range (0–18 years) or specifically around the moment of local anaesthetic administration—plausibly the point of greatest procedural stress—neither of which was addressed by the included evidence.

A related gap concerns outcome measurement: as detailed in Section 3.5, no instrument was used consistently across studies, populations, or age groups, and a future core outcome set should explicitly accommodate developmental variation and the caregiver–child relationship rather than assuming a single instrument suite suits early childhood through adolescence.

Considerable methodological heterogeneity—in intervention modality, intensity, duration, and individualisation, and in outcome assessment—limited direct comparison, precluded quantitative synthesis for most categories, and contributed to downgrading across several GRADE domains. A consensus core outcome set would substantially improve comparability, evidence synthesis, and future guideline development.

Clinically, the evidence supports cautious consideration of SADE and audiovisual distraction (including VR) as adjuncts within an individualised behaviour-management strategy, and a more tentative, lower-certainty consideration of music therapy given its narrower direct trial base following this revision, rather than universally recommended first-line approaches; moderate-to-low certainty indicates the true effect may differ meaningfully from current estimates, and implementation should account for individual patient characteristics, sensory preferences, developmental status, treatment complexity, resources, and clinician expertise. These interventions appear best positioned as complementary to, rather than replacements for, established behaviour-guidance techniques, particularly for children with pronounced sensory sensitivities.

## 5 Limitations

Neurodevelopmental status was not used as a formal stratification variable, limiting population-specific interpretation. Two studies originally classified as GRADE-eligible were reclassified as narrative-only during preparation of this revision: McBeain et al. (23), which does not meet the randomised/controlled-trial threshold, and Chen and Hawkins (40), a single-arm pilot without a concurrent comparator group (Sections 2.2, 3.4.3, 3.4.5). One study originally included (Cruz-Fierro et al., 35) was subsequently excluded on discovering its sample comprised adults rather than children; all three changes are reflected throughout Section 3 and Tables 1–3. All included studies were ultimately published in English despite no formal language restriction, introducing potential language bias. PROSPERO registration (CRD420261456366) was completed concurrently with, rather than prior to, manuscript submission, so methodological decisions cannot be independently verified as pre-specified. The evidence was further limited by methodological heterogeneity, small sample sizes, inconsistent adverse-event reporting, and limited long-term follow-up; although no adverse events were reported, surveillance was not systematic, and this should not be read as confirmed safety. Formal publication-bias assessment was feasible for only two modalities given limited study numbers elsewhere.

**Table 3:** Clinical recommendations by evidence certainty.

Modality	Evidence base	GRADE certainty	Clinical recommendation	Research priority
Sensory-adapted dental environments (SADE)	3 GRADE-eligible RCTs/controlled trials (of 9 total)	Moderate	Can be considered as part of routine or special-care management, particularly for children with ASD or Down syndrome	Multicentre RCTs with standardised SADE component sets (lighting, sound, tactile/proprioceptive input)
Audiovisual distraction/virtual reality	8 GRADE-eligible RCTs (of 10 total)	Moderate	Suitable adjunct for procedural anxiety and pain management across neurotypical and neurodivergent children	Long-term and multi-session studies; standardised devices/content and comparator conditions
Music therapy and auditory distraction	1 GRADE-eligible RCT (of 4 total) + corroborating secondary meta-analyses	Low	Reasonable adjunct, particularly for pre-procedural anxiety reduction, but recommendation is more tentative pending additional primary trials	Additional RCTs with concurrent comparator groups; standardised protocols for format, timing, duration and delivery method
Aromatherapy	4 GRADE-eligible RCTs	Low	Insufficient evidence to support as a stand-alone strategy; may be considered as part of a combined sensory approach	Large RCTs isolating the independent olfactory-only effect; systematic adverse-event monitoring
Weighted blankets/deep-pressure interventions	2 GRADE-eligible RCTs (of 4 total)	Low	May be considered as an adjunct in selected patients (e.g., intellectual/developmental disability, sedation visits)	Larger RCTs across clinical contexts; standardised weight/duration protocols; prospective safety monitoring
Social stories	1 GRADE-eligible RCT (of 3 total)	Low	Consider for preparatory/preventive use in ASD or special healthcare needs; not established for intra-procedural anxiety	RCTs evaluating use during active dental treatment; longer-term caries and attendance outcomes
Video modelling	1 GRADE-eligible RCT (of 4 total)	Low	Consider as a preparatory tool for non-invasive care, particularly in children with ASD	RCTs isolating video modelling from combined interventions (e.g., systematic desensitisation)
Visual pedagogy/visual schedule systems	2 GRADE-eligible RCTs (of 5 total)	Low	Consider for preventive/preparatory procedures in children with ASD	Controlled trials extending evaluation to restorative and other invasive procedures
Emerging modalities (social robotics, Snoezelen, yoga, animal-assisted therapy)	2 GRADE-eligible RCTs (of 4 total, across distinct sub-modalities)	Very low	Insufficient evidence for routine recommendation; regard as experimental/exploratory	Adequately powered, multicentre RCTs with standardised outcome measures for each sub-modality

Future research should prioritise adequately powered multicentre RCTs with standardised protocols, validated core outcome measures, longer follow-up, and transparent adverse-event reporting. Mechanistic studies using objective physiological measures, and studies stratifying by sensory phenotype rather than diagnostic category, may clarify how individual interventions act and for whom; implementation and cost-effectiveness studies will be needed to translate these findings into routine paediatric dental practice across diverse settings. Table 3 summarises clinical recommendations and research priorities for each modality, framed by their GRADE certainty ratings.

## 6 Conclusions

This review provides a GRADE-informed synthesis of sensory-based non-pharmacological interventions for reducing dental anxiety and improving behavioural cooperation in paediatric patients. Across 47 included studies spanning nine sensory intervention categories, these interventions were consistently associated with improvements in behavioural, psychological, and physiological outcomes, though certainty of evidence ranged from moderate to very low, indicating that confidence in these findings varies by modality.

Current evidence supports cautious clinical consideration of sensory-adapted dental environments and audiovisual distraction (including virtual reality) as adjuncts to established behaviour-guidance strategies within an individualised, patient-centred approach, with music therapy offering a promising but lower-certainty adjunct pending additional primary trials with concurrent comparator groups. While the observed physiological changes are compatible with modulation of autonomic nervous system activity, the underlying mechanisms remain incompletely understood, and no definitive mechanistic conclusions can be drawn from the available evidence.

Future high-quality multicentre randomised controlled trials employing standardised protocols, consensus outcome measures, rigorous adverse-event reporting, and longer-term follow-up are needed to strengthen the evidence base and inform clinical practice guidelines.

## Data Availability

The original contributions presented in the study are included in the article/Supplementary Material, further inquiries can be directed to the corresponding author.
