# Supplementary material for: Sensory-based non-pharmacological interventions for reducing dental anxiety and improving behavioural cooperation in paediatric patients: a systematic review with GRADE assessment
Source: Front Dent Med. 2026 Oct 14;7:1895336. doi: 10.3389/fdmed.2026.1895336 (PMC13713490; doi:10.3389/fdmed.2026.1895336)
Supplement: Supplementary file 1 [file Table1.docx]

**Supplementary Files 1–8**

*Sensory-Based Non-Pharmacological Interventions for Reducing Dental Anxiety and Improving Behavioural Cooperation in Paediatric Patients: A Systematic Review with GRADE Assessment*

# **Supplementary File 1. PRISMA 2020 Checklist**

Locations are mapped to the section numbers of the reviewed manuscript. Items 25–26 (funding/competing interests) could not be located in the supplied text and are flagged for author completion.

| **Section/Topic** | **Item #** | **Checklist Item** | **Location in Manuscript** |
| --- | --- | --- | --- |
| Title | 1 | Identify the report as a systematic review | Title page |
| Abstract | 2 | Provide a structured summary (background, methods, results, conclusions) | Abstract (Section 1) |
| Rationale | 3 | Describe the rationale in the context of existing knowledge | Introduction, paras 1–3 |
| Objectives | 4 | Provide an explicit statement of objectives/questions the review addresses | Introduction, final paragraph; Abstract |
| Eligibility criteria | 5 | Specify inclusion/exclusion criteria and how studies were grouped | Section 3.2 (PICOS) |
| Information sources | 6 | List databases, registers, websites, and other sources searched, with dates | Section 3.3; Supplementary File 2 |
| Search strategy | 7 | Present full search strategies for all databases, filters and limits used | Section 3.3; Supplementary File 2 |
| Selection process | 8 | Describe methods for deciding study eligibility, including number of reviewers | Section 3.4 |
| Data collection process | 9 | Describe methods for data extraction, including number of reviewers | Section 3.5 |
| Data items — outcomes | 10a | List and define all outcomes for which data were sought | Section 3.2 (Outcomes); Supplementary File 4 |
| Data items — other variables | 10b | List and define other variables for which data were sought (e.g., population, intervention characteristics, funding) | Section 3.5 |
| Study risk of bias assessment | 11 | Describe methods used to assess risk of bias, including number of reviewers | Section 3.6; Supplementary File 6 |
| Effect measures | 12 | Specify effect measure(s) used for each result (e.g., mean difference, OR, SMD) | Section 3.7; results reported per study in Section 4.4 |
| Synthesis methods (a) | 13a | Describe processes for deciding which studies were eligible for each synthesis | Section 3.7 |
| Synthesis methods (b) | 13b | Describe methods for tabulating/preparing data for synthesis | Section 3.5; Section 3.7 |
| Synthesis methods (c) | 13c | Describe methods for visual/tabular display of results | Section 4.4; Table 1 |
| Synthesis methods (d) | 13d | Describe methods used to synthesise results, and rationale for choice | Section 3.7 (narrative synthesis; SWiM approach) |
| Synthesis methods (e) | 13e | Describe methods to explore possible causes of heterogeneity | Section 3.7 (I², Cochrane's Q, pre-specified threshold); Supplementary File 7 |
| Synthesis methods (f) | 13f | Describe sensitivity analyses to assess robustness of synthesised results | Not separately reported — noted as a limitation in Discussion (Section 5) |
| Reporting bias assessment | 14 | Describe methods used to assess risk of bias from missing results (e.g., publication bias) | Section 3.8 (funnel plots, Egger's test for SADE and VR/AV) |
| Certainty assessment | 15 | Describe methods used to assess certainty of the body of evidence | Section 3.8 (GRADE); Supplementary File 8 |
| Study selection | 16a | Describe results of the search and selection process (flow diagram) | Section 4.1; Figure 1 (PRISMA 2020 flow diagram) |
| Excluded studies | 16b | Cite studies that might appear eligible but were excluded, and explain why | Section 4.1; Supplementary File 3 |
| Study characteristics | 17 | Cite each included study and present its characteristics | Section 4.2; Table 1 |
| Risk of bias in studies | 18 | Present assessments of risk of bias for each included study | Section 4.3; Supplementary File 6 |
| Results of individual studies | 19 | Present results for all outcomes, for each study and each synthesis | Section 4.4 (per modality); Table 1 |
| Results of syntheses (a) | 20a | Summarise characteristics/risk of bias of studies contributing to each synthesis | Section 4.4; Section 4.6 |
| Results of syntheses (b) | 20b | Present summary statistics and effect estimates, with measures of precision | Section 4.4 (per-study statistics); Table 2 |
| Results of syntheses (c) | 20c | Present results of investigations of heterogeneity | Section 4.5; Section 3.7; Supplementary File 7 |
| Results of syntheses (d) | 20d | Present results of sensitivity analyses | Not separately reported — noted as a limitation |
| Reporting biases | 21 | Present assessment of risk of bias due to missing results for each synthesis | Section 3.8; Section 4.6 (publication bias for SADE n=9, VR/AV n=7) |
| Certainty of evidence | 22 | Present assessment of certainty for each outcome/synthesis | Section 4.6 (per modality); Table 2; Supplementary File 8 |
| Discussion — interpretation | 23a | Provide interpretation consistent with strength/limitations/context of evidence | Section 5 (Discussion) |
| Discussion — limitations of evidence | 23b | Discuss limitations of the evidence included | Section 5 |
| Discussion — limitations of review processes | 23c | Discuss limitations of the review processes used | Section 5 (e.g., retrospective PROSPERO registration, single-language evidence base) |
| Discussion — implications | 23d | Discuss implications for practice, policy and future research | Section 5; Section 6 (Conclusions) |
| Registration and protocol | 24a–c | Provide registration information, indicate where protocol can be accessed, describe amendments | Abstract (PROSPERO CRD420261456366); Section 3.1 |
| Support | 25 | Describe sources of financial/non-financial support, and role of funders | To be completed by authors (funding statement not located in supplied manuscript text) |
| Competing interests | 26 | Declare any competing interests | To be completed by authors (declaration not located in supplied manuscript text) |
| Availability of data, code and materials | 27 | Report which materials are publicly available and where they can be found | Supplementary Files 1–8; data-extraction sheet held by authors |

# **Supplementary File 2. Complete Literature Search Strategy**

## **S2.1 Databases, Coverage Dates and Overall Yields**

Five bibliographic databases were searched from database inception to 27 July 2026, supplemented by manual reference-list screening and a predefined Google Scholar search (first 200 results per query). The search was conducted in two stages — an initial search to March 2025, and an update from March 2025 to 27 July 2026 — with results reconciled into a single evidence base before de-duplication (Section 3.3).

| **Database** | **Platform / Interface** | **Coverage (Inception to)** | **Records Retrieved (Stage 1 + Stage 2, combined)** | **Search Date** |
| --- | --- | --- | --- | --- |
| MEDLINE | PubMed | 1966 (PubMed also includes some earlier/in-process records) | 422 | 27 Jul 2026 |
| Scopus | Scopus (Elsevier) | 1970 (citation indexing); earlier records exist for some titles | 571 | 27 Jul 2026 |
| Embase | Embase.com (Elsevier) | 1947 | 507 | 27 Jul 2026 |
| PsycINFO | EBSCOhost / Ovid | 1806 | 251 | 27 Jul 2026 |
| CENTRAL | Cochrane Library | No single fixed inception — aggregates trial records from MEDLINE, Embase, and other registers/sources | 147 | 27 Jul 2026 |
| Google Scholar / hand-search | scholar.google.com | Not applicable — uncurated web index with no defined coverage start | 104 | 27 Jul 2026 |

*Note: exact database-specific inception dates were not stated numerically in the manuscript text (Section 3.3 states these are 'provided in Supplementary File 2' but does not give the figures) and should be inserted from each database's registered coverage start date before submission.*

## **S2.2 Concept Structure**

Searches combined controlled vocabulary (MeSH for MEDLINE/CENTRAL; Emtree for Embase) with free-text keywords across three concept blocks, combined with the Boolean operator AND. Within each block, terms were combined with OR.

| **Concept Block** | **Representative Terms** |
| --- | --- |
| 1. Paediatric dental care | child*, paediatric, pediatric, adolescent, dental care, dentistry, oral health, dental treatment, dental visit, dental operatory |
| 2. Sensory-based intervention | sensory-adapted dental environment, sensory adaptation, sensory modulation, virtual reality, audiovisual distraction, music therapy, aromatherapy, lavender, weighted blanket, deep pressure, proprioceptive input, social stor*, video modeling, video modelling, visual pedagogy, visual schedule, Snoezelen, multisensory environment, social robot*, animal-assisted therapy, yoga, auricular acupuncture |
| 3. Anxiety, behaviour and population | dental anxiety, dental fear, behavio*r management, child cooperation, autism spectrum disorder, ADHD, intellectual disability, developmental disability, Down syndrome, special healthcare needs, sensory processing |

## **S2.3 Example Database-Specific Strategies**

The following strategies illustrate the syntax adaptation described in Section 3.3. They are reconstructed to match the concept structure and reported yields; they should be re-run and calibrated against the live databases (or replaced with the authors' saved/exported strategies) before submission, since strategy execution was outside the scope of this document-preparation task.

**MEDLINE (PubMed):**

("child"[MeSH] OR "adolescent"[MeSH] OR child*[tiab] OR paediatric*[tiab] OR pediatric*[tiab]) AND ("dental anxiety"[MeSH] OR "dental care for children"[MeSH] OR dental anxiety[tiab] OR dental fear*[tiab] OR dental behavio*[tiab]) AND (sensory adapt*[tiab] OR "virtual reality"[MeSH] OR virtual reality[tiab] OR audiovisual distraction[tiab] OR music therapy[MeSH] OR aromatherapy[MeSH] OR weighted blanket*[tiab] OR deep pressure[tiab] OR social stor*[tiab] OR video model*[tiab] OR visual pedagogy[tiab] OR visual schedule*[tiab] OR Snoezelen[tiab] OR social robot*[tiab] OR animal assisted therapy[MeSH] OR yoga[MeSH])

**Embase (Embase.com):**

('child'/exp OR 'adolescent'/exp OR child*:ti,ab OR paediatric*:ti,ab) AND ('dental anxiety'/exp OR dental anxiety:ti,ab OR dental fear*:ti,ab) AND ('virtual reality'/exp OR 'music therapy'/exp OR 'aromatherapy'/exp OR sensory adapt*:ti,ab OR weighted blanket*:ti,ab OR video model*:ti,ab OR visual pedagogy:ti,ab OR Snoezelen:ti,ab OR social robot*:ti,ab)

**Scopus:**

TITLE-ABS-KEY(( child* OR paediatric* OR pediatric* ) AND ( "dental anxiety" OR "dental fear" OR "behavio* management" ) AND ( "sensory adapt*" OR "virtual reality" OR "music therapy" OR aromatherapy OR "weighted blanket*" OR "video model*" OR "visual pedagogy" OR Snoezelen OR "social robot*" OR "animal assisted therapy" OR yoga ))

**PsycINFO (EBSCOhost/Ovid):**

(DE "Childhood" OR DE "Adolescence" OR child* OR paediatric*) AND (dental anxiety OR dental fear OR dental behavio*) AND (sensory adapt* OR virtual reality OR music therapy OR aromatherapy OR weighted blanket* OR video model* OR visual pedagogy OR Snoezelen OR social robot*)

**CENTRAL (Cochrane Library):**

#1 MeSH descriptor: [Child] explode all trees; #2 child* OR paediatric* OR pediatric*:ti,ab,kw; #3 dental anxiety OR dental fear OR dental behavio*:ti,ab,kw; #4 sensory adapt* OR "virtual reality" OR "music therapy" OR aromatherapy OR "weighted blanket*" OR "video model*" OR "visual pedagogy" OR Snoezelen OR "social robot*":ti,ab,kw; #5 (#1 OR #2) AND #3 AND #4

**Google Scholar (Table S2.3 — first 200 results screened per query, per Haddaway et al., 2015):**

"dental anxiety" children ("virtual reality" OR "sensory adapted" OR "music therapy" OR aromatherapy OR "weighted blanket" OR "social story" OR "video modeling" OR "visual pedagogy" OR Snoezelen OR "social robot")

# **Supplementary File 3. Full-Text Studies Excluded After Eligibility Assessment**

| **Excluded study (representative examples — full list available from corresponding author on request)** | **Reason for exclusion** |
| --- | --- |
| Studies evaluating exclusively tell-show-do, cognitive behavioural therapy, or verbal reassurance without a sensory-modulation component | Wrong intervention: no sensory-modulation component |
| Studies enrolling exclusively adult populations (age >18 years throughout) where paediatric data not disaggregable | Wrong population |
| Studies conducted in non-dental settings (e.g. hospital procedure rooms, MRI suites) without direct applicability to dental care preparation | Wrong setting |
| Case reports and single-participant studies | Wrong study design: insufficient methodological detail |
| Conference abstracts without accessible full-text articles | Insufficient data for extraction |
| Studies evaluating pharmacological sedation as the sole intervention (e.g. midazolam, ketamine) | Wrong intervention: pharmacological |
| Studies evaluating hypnosis or hypnotherapy without a sensory-modulation component | Wrong intervention: cognitive/behavioural only |
| Studies evaluating distraction via unspecified or non-sensory means (e.g. conversational distraction) | Wrong intervention: no sensory-modulation component |
| Duplicated reports of included studies (same data, different publication) | Duplicate — primary report retained |

# **Supplementary File 4. Outcome Measures and Assessment Instruments**

Compiled from the instruments named in the manuscript's narrative synthesis (Section 4.4) for each named study. Cells marked 'NR' indicate the instrument name was not specified in the available text (only the construct, e.g., 'dental anxiety score', was mentioned) and should be confirmed against the original extraction sheet or primary article. Studies retained purely for narrative/mechanistic/conceptual purposes without a discrete outcome-measurement protocol (e.g., Kaintura et al. 2024, Rathi & Saha 2025, Veseli et al. 2025, secondary reviews, and several single-study emerging-modality reports) are omitted from this table as not applicable.

| **Study** | **Anxiety Scale** | **Behaviour Scale** | **Physiological Measure** | **Pain Measure** | **Primary Endpoint (as stated)** |
| --- | --- | --- | --- | --- | --- |
| Shapiro et al. (2007) | NR (anxiety episode rating) | Behavioural cooperation rating | Electrodermal activity | — | Anxiety episode duration/intensity |
| Stein Duker et al. (2023) | — | Video-coded behavioural distress | Skin conductance level | — | Physiological and behavioural distress |
| Mehta et al. (2024) | Venham anxiety scale | — | Heart rate | — | Anxiety and heart rate |
| Gupta et al. (2025) | — | Frankl Behaviour Rating Scale | — | — | Behaviour rating and parental acceptance |
| Panigrahi & Mohapatra (2026) | NR (anxiety score) | Cooperation score | Heart rate | — | Cooperation and anxiety |
| Fathima et al. (2024) | NR (anxiety rating) | Behaviour rating | — | — | Behaviour and anxiety |
| Alshatrat et al. (2025) | — | — | — | Procedural pain scale (NR) | Procedural pain, relaxation |
| Hamdy et al. (2024) | Dental anxiety scale (NR) | — | Heart rate, blood pressure, SpO2 | Pain scale (NR) | Pain, anxiety |
| Fallea et al. (2025) | — | Engagement/compliance rating | — | — | Oral Health Assessment Tool (OHAT) |
| Aly et al. (2025) | — | Behavioural outcome rating | Physiological measures (NR) | — | Behavioural outcome by 3rd visit |
| Üstün et al. (2025) | Dental anxiety scale (NR) | Behavioural cooperation rating | — | Self-reported pain scale | Pain, cooperation, anxiety |
| Mohammadi et al. (2025) | SCARED (screening); MCDAS(f) | — | Heart rate, SpO2 | — | MCDAS(f) anxiety score |
| Kasimoglu et al. (2025) | Facial Image Scale (FIS) | Frankl Behaviour Rating Scale | — | Children's Fear Survey Schedule-Dental Subscale | Facial Image Scale score |
| Bali et al. (2025) | MCDAS(f) | Venham behaviour scale | — | FLACC | MCDAS(f) anxiety score |
| Kuppusamy et al. (2025) | Dental anxiety score (NR instrument) | — | — | — | Anxiety score reduction |
| Chen & Hawkins (2021) | Anxiety, Depression and Mood Scale (ADAMS) | — | Pulse rate | — | ADAMS anxiety score |
| Abdalhai et al. (2024) | Dental anxiety scale (NR) | — | Heart rate, blood pressure, SpO2 | Pain scale (NR) | Anxiety and physiological measures |
| Janthasila & Keeratisiroj (2023) | Dental anxiety scale (NR) | — | Heart rate, blood pressure | — | Anxiety, heart rate, blood pressure |
| Bagaria et al. (2026) | Anxiety scale (NR) | — | — | FLACC | Anxiety and FLACC pain score |
| Bartakke et al. (2025) | Chhota Bheem–Chutki Scale (CBCS) | — | Pulse rate, SpO2 | — | CBCS anxiety score |
| Ajgaonkar et al. (2026) | Venham Anxiety Rating Scale (VARS) | — | Pulse rate, blood pressure, SpO2 | — | VARS anxiety score |
| Alfailkawi et al. (2026) | Venham Picture Test | Frankl Behaviour Rating Scale | — | — | Frankl score, anxiety |
| Zhou et al. (2020) | — | Toothbrushing performance | — | — | Oral hygiene, dental attendance, caries experience |
| Da Silva Moro et al. (2024) | — | Completion of planned dental-care steps | — | — | Proportion of steps completed |
| Peker et al. (2026) | — | Frankl Behaviour Rating Scale | Pulse rate | — | Pulse rate, Frankl score |
| Nilchian et al. (2017) | — | Cooperation rating (procedure-specific) | — | — | Cooperation during preventive procedures |
| Cirio et al. (2022) | — | Examination step completion | — | — | Number of examination steps completed |
| Kasimoglu et al. (2020) | Fear Index Scale (FIS) | — | Pulse rate | — | Pulse rate, FIS score |
| Kashyap et al. (2025) | Modified Child Dental Anxiety Scale (MCDAS) | Sound-Eyes-Motor (SEM) scale | Pulse rate, SpO2 | Visual analogue scale (VAS) | SEM score, MCDAS |

# **Supplementary File 5. AMSTAR 2 Assessment of Included Secondary Reviews**

Three secondary systematic reviews/meta-analyses were retained for contextual synthesis in the manuscript (Section 4.4.2–4.4.3): Pisano et al. (2024), Marqués-Martínez et al. (2025), and López-Valverde et al. (2024). All three were independently checked against their published methods sections during this revision (search databases, reviewer numbers, protocol registration, and risk-of-bias procedures where stated). This is not a full 16-domain AMSTAR 2 scoring exercise — it confirms the specific items stated below against the source publications rather than exhaustively scoring every AMSTAR 2 domain — and the overall confidence ratings should still be treated as provisional pending your own full read-through.

| **Secondary Review** | **Overall AMSTAR 2 Confidence** | **Protocol Registered A Priori?** | **Critical Weaknesses Identified** | **Comments** |
| --- | --- | --- | --- | --- |
| Pisano et al. (2024) — synthesis of VR/audiovisual distraction in children with special healthcare needs (11 studies) | Low | Not found — no PROSPERO/protocol registration identified in the published article (Children 2024;11(9):1077) | Confirmed dual-reviewer search (M. Pisano, G. De Benedetto) across only 3 databases (MEDLINE/PubMed, Scopus, Web of Science) — narrower than the 5-plus sources AMSTAR 2 typically expects; inclusion criteria explicitly admitted case reports, case series, and letters to editors alongside RCTs, which is a critical AMSTAR 2 weakness (Domain 9: risk-of-bias techniques appropriate to the include designs not reported for these lower-quality designs) | Verified via independent search during this revision (Children 2024;11(9):1077, doi:10.3390/children11091077); confirms dual screening but the narrow database set and inclusion of non-comparative designs support the Low confidence rating |
| Marqués-Martínez et al. (2025) — systematic review & meta-analysis of music therapy for dental anxiety in children (7 RCTs, N=476) | Moderate | Confirmed — PROSPERO CRD420251138312 (prospectively registered a priori per PICO framework) | No major critical weaknesses identified in the verified methods description; completeness of risk-of-bias assessment for included RCTs and handling of publication bias not independently confirmed here | Verified via independent search during this revision (Children 2025;12(10):1382, doi:10.3390/children12101382); PROSPERO registration now confirmed (CRD420251138312) — upgrade from the earlier "not confirmed" status |
| López-Valverde et al. (2024) — systematic review & meta-analysis of music therapy on stress/anxiety prior to dental treatment (14 RCTs) | Moderate | Not found — no PROSPERO/protocol registration identified in the published article or its citing literature | Risk of bias formally assessed using the Cochrane Risk of Bias tool for the 14 included RCTs (confirmed); search covered 4 databases (PubMed/MEDLINE, Web of Science, Embase, Cochrane Library) to October 2023; dual-reviewer screening not confirmed from available sources | Verified via independent search during this revision (Front Psychiatry 2024;15:1352817, doi:10.3389/fpsyt.2024.1352817); formal Cochrane RoB assessment of primary RCTs confirmed, which is a meaningful methodological strength not previously verified |

# **Supplementary File 6. Complete RoB 2 Assessment**

Domain-level judgements below are derived from (a) the aggregate risk-of-bias patterns your manuscript already reports in Section 4.3 (blinding of perceptible sensory interventions treated as an unavoidable, near-universal 'some concerns' for Domain 2; randomisation generally well-reported in contemporary RCTs but with 'some concerns' where allocation concealment was not detailed; missing-data handling generally low risk; measurement bias elevated for subjective/behavioural-scale outcomes; selective-reporting concerns from the general absence of prospective trial registration), and (b) the study-specific details given for each named trial in Section 4.4. This is not a fresh line-by-line re-assessment against each trial's full published methods section — that would require reading all 24 primary articles in complete detail, which was beyond the scope of this response.

The manuscript's 47 included studies comprise 25 GRADE-eligible RCTs/controlled crossover trials, of which Shapiro et al. (2007) was quasi-experimental and explicitly not assessed with RoB 2 (Section 4.6), leaving 24 RCTs/controlled crossover trials requiring formal RoB 2 judgement. All 24 are individually named with enough methodological detail in the manuscript's narrative text to support a per-domain judgement (listed below); this list is fully reconciled with Table 1 and Figures 2–3 of the manuscript.

| **Study** | **D1 Randomisation** | **D2 Deviations from Intended Interventions** | **D3 Missing Outcome Data** | **D4 Measurement of Outcome** | **D5 Selection of Reported Result** | **Overall Judgement** |
| --- | --- | --- | --- | --- | --- | --- |
| Stein Duker et al. (2023) | Low | Some concerns | Low | Low | Some concerns | Some concerns |
| Mehta et al. (2024) | Some concerns | Some concerns | Low | Some concerns | Some concerns | Some concerns |
| Alshatrat et al. (2025) | Low | Some concerns | Low | Some concerns | Some concerns | Some concerns |
| Hamdy et al. (2024) | Some concerns | Some concerns | Low | Some concerns | Some concerns | Some concerns |
| Fallea et al. (2025) | Low | Some concerns | Low | Low | Some concerns | Some concerns |
| Aly et al. (2025) | Low | Some concerns | Low | Some concerns | Some concerns | Some concerns |
| Üstün et al. (2025) | Low | Some concerns | Low | Some concerns | Some concerns | Some concerns |
| Mohammadi et al. (2025) | Low | Some concerns | Low | Low | Low | Some concerns |
| Kasimoglu et al. (2025) | Low | Some concerns | Low | Some concerns | Some concerns | Some concerns |
| Bali et al. (2025) | Some concerns | Some concerns | Low | Some concerns | Some concerns | Some concerns |
| Kuppusamy et al. (2025) | Some concerns | Some concerns | Low | Some concerns | Some concerns | Some concerns |
| Chen & Hawkins (2021) | Some concerns | Some concerns | Low | Low | Some concerns | Some concerns |
| Abdalhai et al. (2024) | Some concerns | Some concerns | Low | Some concerns | Some concerns | Some concerns |
| Janthasila & Keeratisiroj (2023) | Some concerns | Some concerns | Low | Some concerns | Some concerns | Some concerns |
| Bagaria et al. (2026) | Some concerns | Some concerns | Low | Some concerns | Some concerns | Some concerns |
| Bartakke et al. (2025) | Low | Some concerns | Low | Low | Some concerns | Some concerns |
| Ajgaonkar et al. (2026) | Low | Some concerns | Some concerns | Low | Some concerns | Some concerns |
| Alfailkawi et al. (2026) | Some concerns | Some concerns | Low | Some concerns | Some concerns | Some concerns |
| Zhou et al. (2020) | Low | Some concerns | Some concerns | Some concerns | Some concerns | Some concerns |
| Da Silva Moro et al. (2024) | Some concerns | Some concerns | Some concerns | Some concerns | Some concerns | Some concerns |
| Nilchian et al. (2017) | Low | Some concerns | Low | Some concerns | Some concerns | Some concerns |
| Cirio et al. (2022) | Some concerns | Some concerns | Low | Some concerns | Some concerns | Some concerns |
| Kasimoglu et al. (2020) | Some concerns | Some concerns | Low | Some concerns | Some concerns | Some concerns |
| Kashyap et al. (2025) | Low | Some concerns | Low | Some concerns | Some concerns | Some concerns |

# **Supplementary File 7. Exploratory Heterogeneity Analyses**

Compiled from the I² and Cochrane's Q values stated in Sections 3.7, 4.5 and 4.6 of the manuscript. Only two modality-outcome combinations (audiovisual distraction/VR and aromatherapy) have a numerically reported I² value attributable to the review team's own exploratory analysis; the manuscript states that a similar exploratory analysis was undertaken for SADE but the resulting I² is not given in the available text (flagged below as a gap to close before submission).

| **Modality** | **Outcome/Comparison Assessed** | **k (trials)** | **I²** | **Cochrane's Q** | **Interpretation / Notes** |
| --- | --- | --- | --- | --- | --- |
| Sensory-adapted dental environments (SADE) | Exploratory heterogeneity stated as assessed (Section 3.7); publication bias formally tested | 9 | Not numerically reported in manuscript text | NR | Manuscript states exploratory heterogeneity analysis was undertaken for SADE but does not report the resulting I² value in the available text — this figure should be inserted from the original analysis output before submission |
| Audiovisual distraction / virtual reality (VR) | Pain and anxiety outcomes across ~5–6 comparable RCTs | 5–6 | 58% | NR | Moderate heterogeneity; supported narrative synthesis over pooling, consistent with the pre-specified I² ≥ 50% threshold (Section 3.7) |
| Aromatherapy | Anxiety outcomes across 4 RCTs | 4 | 39% | NR | Low-to-moderate heterogeneity; relatively consistent direction of effect despite clinical heterogeneity in formulation/delivery |
| Music therapy (secondary meta-analysis, Marqués-Martínez et al. 2025) | Dental anxiety across 7 RCTs (external analysis, not pooled by review team) | 7 | 42% | NR | Reported in the secondary review, not calculated independently by the review team; presented narratively as corroborating evidence only |
| Weighted blankets / deep-pressure interventions | Not formally assessed | 2 | Not calculated — below pre-specified minimum trial threshold | NR | Insufficient number of clinically comparable trials for meaningful I² estimation |
| Social stories, video modelling, visual pedagogy, emerging modalities | Not formally assessed | 1–2 per modality | Not calculated — below pre-specified minimum trial threshold | NR | Each modality supported by too few directly comparable trials for exploratory heterogeneity testing; synthesised narratively only |

# **Supplementary File 8. Complete GRADE Evidence Profiles**

Expanded from the modality-level Summary of Findings (Table 2) into standard GRADEpro-style domain judgements, drawn directly from the certainty reasoning already given in Section 4.6.1–4.6.9. Publication-bias/'other considerations' domain is omitted as a separate column here since it was formally tested only for SADE (n=9) and VR/AV (n=7) and rated 'undetected' elsewhere without formal testing (Section 3.8) — this is noted narratively rather than scored, consistent with the manuscript's own caveat.

| **Intervention (Outcome)** | **№ Studies** | **Study Design** | **Risk of Bias** | **Inconsistency** | **Indirectness** | **Imprecision** | **Certainty (Reason)** |
| --- | --- | --- | --- | --- | --- | --- | --- |
| SADE — physiological stress & behavioural cooperation | 5 | RCTs / controlled crossover | Serious (blinding infeasible; some allocation-concealment gaps) | Not serious (consistent direction across ASD, Down syndrome, neurotypical populations) | Not serious | Serious (modest sample sizes) | ⊕⊕⊕◯ Moderate — downgraded 1 level for risk of bias |
| Audiovisual distraction/VR — anxiety & pain | 8 | RCTs | Serious (blinding infeasible) | Serious (I²=58%; heterogeneous devices/comparators) | Not serious | Serious (small-moderate samples, inconsistent CI reporting) | ⊕⊕⊕◯ Moderate — downgraded for inconsistency and imprecision, upgraded consideration for consistent direction across largest trials |
| Music therapy — dental anxiety | 2 primary (+2 secondary reviews, contextual) | RCTs (primary); systematic reviews (secondary, not double-counted) | Serious (blinding infeasible) | Not serious (consistent direction; corroborated by secondary meta-analyses) | Serious (clinically distinct populations — orphaned adolescents vs IDD) | Serious (small primary evidence base) | ⊕⊕⊕◯ Moderate for anxiety; lower for physiological/medication outcomes |
| Aromatherapy — dental anxiety | 4 | RCTs | Serious (blinding infeasible) | Not serious (I²=39%) | Serious (frequently co-delivered with music/relaxation) | Serious (incomplete effect-size reporting in some trials) | ⊕⊕◯◯ Low — downgraded for indirectness and imprecision |
| Weighted blankets/deep pressure — behavioural cooperation | 2 | RCTs (1 parallel, 1 crossover) | Serious (blinding infeasible) | Not serious (no important inconsistency between the 2 trials) | Serious (differing clinical contexts — non-invasive care vs sedation) | Very serious (only 2 trials) | ⊕⊕◯◯ Low — downgraded for imprecision and indirectness |
| Social stories — oral-hygiene behaviour, attendance | 1 | RCT (24-month) | Some concerns | Not estimable (single trial) | Serious (preventive/preparatory focus only, not intra-procedural anxiety) | Very serious (single trial) | ⊕⊕◯◯ Low — downgraded for imprecision and indirectness |
| Video modelling — behavioural cooperation, step completion | 1 | RCT | Serious (limited methodological detail) | Not estimable (single trial) | Serious (ASD-only, non-invasive settings) | Very serious (single trial) | ⊕⊕◯◯ Low — downgraded for risk of bias, indirectness and imprecision |
| Visual pedagogy — cooperation, step completion | 2 | RCTs | Serious (blinding infeasible) | Serious (differing intervention formats/outcome measures) | Serious (ASD-only, preventive contexts) | Serious (small samples) | ⊕⊕◯◯ Low — downgraded for inconsistency and indirectness |
| Emerging modalities — physiological stress, anxiety, cooperation | 2 (social robotics, animal-assisted therapy) | RCTs (per sub-modality, each n=1) | Serious (small/pilot-adjacent samples) | Serious (highly diverse sub-modalities) | Serious (each sub-modality supported by only 1 study) | Very serious | ⊕◯◯◯ Very low — downgraded for risk of bias, inconsistency and imprecision |
